# Comparing syngeneic and autochthonous models of breast cancer to identify tumor immune components that correlate with response to immunotherapy in breast cancer

**DOI:** 10.1186/s13058-021-01448-1

**Published:** 2021-08-05

**Authors:** Jessica Castrillon Lal, Madeline G. Townsend, Anita K. Mehta, Madisson Oliwa, Eric Miller, Alaba Sotayo, Emily Cheney, Elizabeth A. Mittendorf, Anthony Letai, Jennifer L. Guerriero

**Affiliations:** 1 Breast Tumor Immunology Laboratory, Susan F. Smith Center for Women’s Cancers, Dana-Farber Cancer Institute, Boston, MA USA; 2 Department of Medical Oncology, Dana-Farber Cancer Institute, 450 Brookline Avenue, Boston, MA 02215 USA; 3 Genomic Medicine Institute, Lerner Research Institute, Cleveland Clinic, Cleveland, OH 44195 USA; 4 Division of Breast Surgery, Department of Surgery, Brigham and Women’s Hospital, Boston, MA 02115 USA; 5 Nanostring Technologies, Seattle, WA USA; 6 Breast Oncology Program, Dana-Farber/Brigham and Women’s Cancer Center, Boston, MA USA; 7 Ludwig Center for Cancer Research at Harvard, Harvard Medical School, Boston, MA USA

**Keywords:** Breast cancer, Immunotherapy, Immune checkpoint blockade, Syngeneic tumor models, Preclinical mouse models, Jessica Castrillon Lal and Madeline G. Townsend contributed equally to this work

## Abstract

**Background:**

The heterogeneity of the breast tumor microenvironment (TME) may contribute to the lack of durable responses to immune checkpoint blockade (ICB); however, mouse models to test this are currently lacking. Proper selection and use of preclinical models are necessary for rigorous, preclinical studies to rapidly move laboratory findings into the clinic.

**Methods:**

Three versions of a common syngeneic model derived from the MMTV-PyMT autochthonous model were generated by inoculating 1E6, 1E5, or 1E4 cells derived from the MMTV-PyMT mouse into wildtype recipient mice. To elucidate how tumor latency and TME heterogeneity contribute to ICB resistance, comprehensive characterization of the TME using quantitative flow-cytometry and RNA expression analysis (NanoString) was performed. Subsequently, response to ICB was tested. These procedures were repeated using the EMT6 breast cancer model.

**Results:**

The 3 syngeneic versions of the MMTV-PyMT model had vastly different TMEs that correlated to ICB response. The number of cells used to generate syngeneic tumors significantly influenced tumor latency, infiltrating leukocyte populations, and response to ICB. These results were confirmed using the EMT6 breast cancer model. Compared to the MMTV-PyMT autochthonous model, all 3 MMTV-PyMT syngeneic models had significantly more tumor-infiltrating lymphocytes (TILs; CD3^+^, CD4^+^, and CD8^+^) and higher proportions of PD-L1-positive myeloid cells, whereas the MMTV-PyMT autochthonous model had the highest frequency of myeloid cells out of total leukocytes. Increased TILs correlated with response to anti-PD-L1 and anti-CTLA-4 therapy, but PD-L1expression on tumor cells or PD-1 expression of T cells did not.

**Conclusions:**

These studies reveal that tumor cell number correlates with tumor latency, TME, and response to ICB. ICB-sensitive and resistant syngeneic breast cancer models were identified, in which the 1E4 syngeneic model was most resistant to ICB. Given the lack of benefit from ICB in breast cancer, identifying robust murine models presented here provides the opportunity to further interrogate the TME for breast cancer treatment and provide novel insights into therapeutic combinations to overcome ICB resistance.

**Supplementary Information:**

The online version contains supplementary material available at 10.1186/s13058-021-01448-1.

## Background

The success of immune checkpoint blockade (ICB) in various human cancer types has stimulated interest in its use for treating breast cancer. Current therapy for breast cancer is guided by the molecular pathology of the tumor. Breast cancer is often driven by overactive hormone signaling (estrogen and/or progesterone receptors; ER, PR) or amplification of growth factor response (HER2) and treated with endocrine therapy or HER2-targeted agents. Alternatively, patients can also be treated with classical therapies such as chemotherapy and/or radiation [1–4]. After initial treatment for early-stage disease, approximately 30% of women will eventually develop recurrent advanced or metastatic disease [5]. Almost all who develop metastatic breast cancer will succumb to the disease, highlighting the need for more effective strategies [6].

Advances in understanding tumor-host immune interactions and their role in cancer progression have led to novel therapeutic strategies for cancer. ICB aims to target T cell inhibitory molecules using antibodies against cytotoxic T-lymphocyte associated protein 4 (CTLA-4) and programmed cell death protein-1 (PD-1) as well as its ligand (PD-L1), which play an important role in central and peripheral immune tolerance. ICB reinvigorates anti-tumor immune responses by inhibiting negative interactions between T cells and antigen-presenting cells (APCs) or tumor cells in several cancer types [7, 8]. In 2011, the first ICB agent, ipilimumab, a human monoclonal antibody targeting CTLA-4, was approved by the FDA for treatment of metastatic melanoma based on significant improvement in overall survival in a randomized, double-blinded phase III study [9]. Importantly, ipilimumab has doubled 10-year survival for metastatic melanoma compared with historical data [10–12]. Antibodies targeting PD-1 and PD-L1 have also shown a durable clinical response in melanoma, as well as renal cell carcinoma, non-small cell lung cancer, and bladder cancer [13–20]. To date, such responses have led to FDA approval of eight ICB therapies across more than 20 cancer types and two tissue-agnostic conditions [21–23]. Because their effector pathways are distinct, the combination of CTLA-4 and PD-1/PD-L1 therapy can provide an enhanced response [24, 25]. The combination has been FDA approved for melanoma [26], renal cell carcinoma [27], non-small cell lung cancer [28], and colorectal cancer [29].

Breast cancer has a lower mutational burden compared to other types of cancer, which may explain the lack of efficacy in response to ICB [30, 31]. Despite this generalization, triple-negative breast cancer (TNBC) has demonstrated some benefit from ICB therapy, albeit not achieving the response rates demonstrated in melanoma and lung cancer. While pembrolizumab (anti-PD-1) showed promising activity as a single agent against advanced or metastatic TNBC in the KEYNOTE-012 (NCT01848834) and KEYNOTE-086 (NCT02447003) clinical trials, in the randomized, Phase III KEYNOTE-119 (NCT02555657) clinical trial, there was no improvement in overall survival compared to single-agent chemotherapy in metastatic TNBC [32]. Benefit from ICB therapy has been observed in patients treated in the first-line setting and/or in patients whose tumors or immune cells express PD-L1 [33–35]. For example, in the PCD4989g (NCT01375842) clinical trial, evaluating atezolizumab as a single agent, expression of PD-L1 on 1% or greater of immune infiltrating cells was associated with a 12% ORR compared to 0% when there was no expression of PD-L1, and high levels of immune cell infiltration (greater than 10%) were independently associated with higher overall response rate (ORR) and overall survival (OS) [34]. Importantly, there are two FDA-approved ICB therapies for breast cancer, the first that came from the Phase III IMpassion130 (NCT02425891) clinical trial that demonstrated atezolizumab in combination with nab-paclitaxel showed significant extension of median disease-free overall survival compared to nab-paclitaxel alone, from 15.5 to 25 months in patients with 1% or more PD-L1-positive immune cells, where there was no benefit in PD-L1 negative tumors [36, 37]. More recently, pembrolizumab, an anti-PD-1 antibody, in combination with different chemotherapy agents, was also approved for the treatment of locally advanced or metastatic TNBC, based on results from the KEYNOTE-355 trial [38].

The use of PD-L1 as a biomarker for ICB has been rigorously investigated but has raised concerns, including a poor agreement between different antibodies as well as scoring between pathologists [39]. To date, there are 9 FDA approvals for the use of ICB based on a specific PD-L1 threshold and companion diagnostic, with variable thresholds both within and across tumor types using several different assays, including approvals at the following PD-L1 positive percentage thresholds: 1, 5, and 50%. In a recent meta-analysis that examined all approvals of ICB as of April 2019, PD-L1 was predictive in 28.9% of those approvals and was either not predictive (53.3%) or not tested (17.8%) in the remaining approvals [21]. This underscores the need to improve ICV efficacy biomarkers and assess what cells expressing PD-L1 are the most predictive of response. Other predictors of response to ICB include the presence of immune cells. In HER2^+^ and TNBC tumors, immune cells have been shown to correlate with better response to HER2-targeted therapy and chemotherapy, respectively [40]. However, immune cell infiltration has been reported to differ among each subtype of breast cancer [41]. Further work to characterize the TME of breast tumors will provide more opportunities for ICB therapy in these patients.

Mouse models have been instrumental in understanding the molecular mechanisms of oncogenesis and metastasis. Translating in vivo preclinical findings to patients depends on how accurately the mouse model replicates histological markers, biochemical pathways, and genetic aberrations observed in the same human tumor type [42]. In light of the advances in immunotherapy, it is now also necessary to meticulously characterize the TME of preclinical mouse models. Here, we have utilized different numbers of cell inoculum derived from the MMTV-PyMT autochthonous model of breast cancer, in which the polyoma middle T (PyMT) oncogene is driven by the mouse mammary tumor virus (MMTV)-LTR, to generate tumors in wild-type FVB/n mice referred to as 1E6, 1E5, and 1E4, based on the number of cells injected to generate tumors. The MMTV-PyMT model is representative of human breast carcinomas, where several of the same signaling transduction pathways that are commonly disrupted in human breast cancer patients are seen in the MMTV-PyMT model, such as the Src family, Ras, and PI3K kinase pathways [43, 44]. In addition, both innate and adaptive immune cells infiltrate the tumor during tumorigenesis [45, 46]. Macrophages have been shown to play a key role in the development of these tumors, in which CCL2 recruits inflammatory monocytes to facilitate breast tumor growth and metastasis [47]. Additionally, it has been shown that the phenotype is mediated through IL-4 expressing CD4^+^ T cells [46]. A spectrum of macrophage phenotypes have been recognized, ranging from classically activated macrophages (“M1”-like) that are effective in clearing intracellular pathogens and can recruit cytotoxic T lymphocytes to activate adaptive immune responses [48]; to alternatively activated macrophages (“M2”-like), which function to help with parasite clearance, exhibit tissue remodeling capabilities, and promote tumor progression by recruiting T regulatory and Th2 T cell subsets lacking cytotoxic functions [49]. Tumor-associated macrophages (TAMs) likely exhibit features of both M1- and M2-like macrophages but more often exhibit an M2-like phenotype that promotes tumor progression and metastasis by secreting factors that regulate angiogenesis and recruit tumor-suppressive cells such as T regulatory (Treg) cells [50, 51]. In line with the lack of clinical efficacy of ICB in breast cancer, preclinical studies have shown that MMTV-PyMT mice are resistant to ICB monotherapy [45, 52, 53].

With respect to ICB therapy in the clinical care of breast cancer patients, work is urgently needed to better understand if immune cell infiltration, including type, number, and/or phenotype, correlates to responses. Understanding what factors are critical for ICB efficacy in breast cancer will allow careful patient selection and/or catalyze clinical development of novel therapies to distinguish non-responders to responders, improve responses that do occur, surmount acquired resistance to immunotherapy, and identify biomarkers that can more accurately predict durable response. Using the right preclinical model is challenging. While the autochthonous MMTV-PyMT has been described to best represent the human disease [43, 54, 55], there are significant financial and time constraints to using this model. Therefore, researchers have adopted the use of inoculating MMTV-PyMT tumor cells into wild-type mice. With translation to the clinic being of high priority, we sought to determine how the autochthonous and derived syngeneic models align. To accomplish this, we generated three versions of MMTV-PyMT syngeneic models derived from the MMTV-PyMT autochthonous model and performed deep immuno-phenotypic analysis and tested response to ICB. We identify poor concordance between the 1E6 and 1E5 syngeneic models with the autochthonous model. The EMT6 breast cancer model was used as a second model to validate these findings and remarkably had a high correlation with observations made with the MMTV-PyMT syngeneic models. In addition, we identify biomarkers and immune mechanisms that correlate with response to ICB therapy.

## Methods

### Animal husbandry

All experiments used either virgin female Balb/c, NU/J, FVB/NJ, or FVB/N autochthonous mice carrying the polyoma middle T (PyMT) transgene under the control of the mammary tumor virus (MMTV) promoter. The FVB/NJ (001800), Balb/c (000651), and Nude (NU/J; 002448) mice were purchased from Jackson laboratory. All mice were maintained within the Dana-Farer Cancer Center (DFCI), and all experiments were conducted under The Institutional Animal Care and Use Committee (IACUC).

### Generation of syngeneic models

For each experiment, separate batches of tumors were harvested from autochthonous MMTV-PyMT mice (referred to as “inoculum”) using an established protocol [56–59]. Late-stage MMTV-PyMT tumors were harvested, and tumor suspension was either immediately injected into recipient FVB/NJ wild-type mice or frozen for subsequent experiments. Each batch of inoculum was simultaneously injected into wildtype mice using one million (1E6), one hundred thousand (1E5), or ten thousand (1E4) tumor cells. Each experiment was run using the same batch of inoculum, with 3–7 recipient mice per group. This entire protocol was repeated 3 times, starting with a new batch of inoculum for each experiment. The tumor suspension was never cultured. Each experiment was performed with 2–3 different batches of cells harvested from MMTV-PyMT mice. FVB/NJ or nude mice were inoculated with one million (1E6), one hundred thousand (1E5), or ten thousand (1E4) cells in the 4th mammary fat pad to generate syngeneic models. The wild-type mice were age-matched to the autochthonous mice at 10–12 weeks of age. Balb/c mice were implanted with either 1E6 or 1E4 EMT6 tumor cells in the 4th mammary fat pad as described above. Each experimental arm included 4–6 mice per group.

### Tumor digestion

Tumors were extracted and minced and subsequently blended using the gentleMACS Dissociator (Miltenyi Biotec cat. #130-093-235). MACS Miltenyi Tumor Dissociation Kit for mouse (Miltenyi Biotec cat. #130-096-730) was used for further enzymatic digestion according to the manufacturer’s protocol. Dissociated tumor cell suspensions were rinsed with RPMI Medium 1640 (Life Technologies cat. # 11875-093), filtered using a 70 μm sterile EASY-strainer^TM^ (Greiner bio-one cat. #542 070), and performed red blood cell lysis using RBC Lysis Solution (Qiagen cat. #158904). The Mouse Tumor Cell Isolation Kit (Miltenyi Biotec: 130-110-187) was used to remove CD45^+^ cells from the inoculum using the Miltenyi Automacs following standard procedures.

### Efficacy studies

Caliper measurements were used to calculate tumor volumes for each mammary tumor using [(length x Width^2^)/2]. MMTV-PyMT mice were enrolled into a study at about 80 days old and when each tumor reached 80–100 mm^3^. Tumors from mammary fat pad numbers 5 and 10 were excluded from the analysis. The sum of the volumes for the MMTV-PyMT autochthonous tumors (1–4 and 5–9) was used and indicated as “total tumor burden.” Syngeneic mice that had tumor measurements ranging between 80 and 100 mm^3^ were enrolled in an experiment. At the indicated time points, animals were euthanized in a CO_2_ chamber before performing a cardiac perfusion with normal saline. The lungs and tumors were removed for analysis.

### Flow cytometry

Tumors were digested as described above, and single cells were re-suspended in a buffer containing 2% FBS and 2mM EDTA (Sigma-Aldrich cat. #E7889) diluted in phosphate-buffered saline (PBS) (Lift Technologies cat. #10010-023). Zombie Aqua^TM^ Fixable Viability Kit (BioLegend cat. #423101) and anti-mouse CD16/CD32 Fc gamma receptor II/III blocking antibody (Affymetrix cat. #14-0161) were diluted in PBS and applied to cells on ice for 20 min in the dark on ice. Cells were washed and incubated with fluorochrome-conjugated antibodies (anti-mouse CD45 Alexa Fluor® 488, clone 30-F11, BioLegend cat. #103122; anti-mouse CD11b Brilliant Violet 711, clone M1/70, BioLegend cat. #101241; anti-mouse CD3 Alexa Fluor® 594, clone 17A2, BioLegend cat. #100240; anti-mouse MHCII Brilliant Violet 421, clone M5/114.15.2, BioLegend cat. #107631; anti-mouse F4/80 Alexa Fluor® 647, clone BM8, BioLegend cat. #123122; anti-mouse CD11c Brilliant Violet 650, clone HL3, BD Biosciences cat. 564079; anti-mouse CD80 Brilliant Violet 605, clone 16-10A1, BioLegend cat. #104729; anti-mouse CD86 PerCP/Cy5.5, clone GL-1, BioLegend cat. #105027; anti-mouse CD40 PE/Cy7, clone 3/23, BioLegend cat. #124621; anti-mouse CD206 PE, clone C068C2, BioLegend cat. #141706; anti-mouse CD8 PE/Cy7, clone 53-6.7, BioLegend cat. #100721; anti-mouse CD4 PE, clone GK1.5, BioLegend cat. #100408; anti-mouse Ly-6G/Ly-6C (GR1) Brilliant Violet 650, clone RB6-8C5, BioLegend cat. #108441; anti-mouse PD-1 Brilliant Violet 421, clone 29F.1A12, BioLegend cat. #135217; anti-mouse PDL-1 PE, clone 10F.9G2, BioLegend cat. #124307, anti-mouse CD-19 Brilliant Violet 605, clone 6D5, BioLegend cat #115539, anti-mouse NK1.1 Alexa Fluor® 647, clone PK136, BioLegend cat. #108719, anti-mouse CD31 Brilliant Violet 421, clone 390, BioLegend cat. #102423, anti-mouse Thy1.1 Brilliant Violet 650, clone OX-7, BioLegend cat. #202533, anti-mouse Thy1.2 PerCP/Cy5.5, clone 53-2.1, BioLegend cat. #140321) in the dark for an hour using the dilution recommended by the manufacturer. Following staining, cells were rinsed with PBS buffer and fixed with 1% paraformaldehyde for 5 min at room temperature. Afterward, cells were rinsed with PBS, re-suspended in PBS buffer, and placed in the dark at 4°C until analysis. Following extracellular staining, cells that obtained an intracellular stain were washed, fixed, and permeabilized using the Foxp3/Transcription Factor Staining Buffer Set Kit (Affymetrix cat. #00-5523) according to the manufacturer’s protocol. Cells were incubated with antibody (anti-mouse Granzyme B Alexa Fluor® 647, clone GB11, BioLegend cat. #515405; anti-mouse FoxP3 PerCP/C5.5, clone R16-715 BD, Biosciences cat. #563902) overnight in the dark at 4°C. The following day, cells were rinsed with PBS and re-suspended with PBS buffer for flow cytometric analysis on the BD LSRFortessa at the Hematologic Neoplasia Flow Cytometry Core of the Dana-Farber Cancer Institute. Five hundred thousand to two million cells were analyzed per sample per mouse using BD FACs Diva Software. Single-color controls were included in the quality control analysis. Total number of mice used per experiment are shown in each graph (1E6=15, 1E5=17, 1E4=14, Tg=16). In two instances, there was one less sample due to technical or experimental error and 1 mouse’s weight was not recorded in the 1E6 group, and therefore, enumeration calculations could not be performed and one tumor sample in the 1E5 group failed on flow cytometry in the intracellular staining panel. Otherwise, all samples were used for analysis. Data analysis and compensation were performed on BD FACS Diva software. The absolute cell number populations were calculated using the equation below:

$$ Absolute\ cell\ population=\frac{\%{cell\ population}_{viable}\ast \left| viable\ cell s\right|\ }{100\ast tumor\ weight} $$

Student’s t tests were performed in Prism version 7 (Graphpad, Inc.), and *P* values are designated as *P < 0.05,**P < 0.01, and ***P < 0.001. All graphs show mean and error bars represent standard error of the mean (s.e.m).

### Dosing

All in vivo experiments were treated with intraperitoneal injections. Mice were treated twice a week with 200 ug of InVivoMAb rat IgG2b isotype control, anti-keyhole limpet hemocyanin (clone LTF-2, BioXcell BE0090), InVivoMab anti-mouse CTLA-4 (clone 9H10, BioXcell BE 0131), and InVivoMab anti-mouse PDL-1 (clone 10F9G2, BioXcell BE0101). Mice were treated until tumors reached 2 cm in one direction. Mouse weight was monitored and recorded weekly. Tumor volumes were measured and plotted as mean total tumor burden ± SEM. Significant differences in tumor fold change were measured by a two-way analysis of variance (ANOVA) multiple comparisons on ranks. The statistical significance of survival curves was assessed using the Kaplan-Meier log-rank analysis. All statistical analysis was performed in Prism version 7 (Graphpad, Inc.). *P* values are designated as *P < 0.05, **P < 0.01, and ***P < 0.001.

### RNA isolation

When syngeneic mouse tumors reached 100 mm^3^, tissue samples were snap-frozen for later processing. Samples were also collected from autochthonous mice with a total tumor burden in the range of 300–600 mm^3^. Tumor specimens of 30 mg were used for RNA isolation using the RNeasy Mini Kit (Qiagen cat. #74104). β-Mercaptoethanol was added to Buffer RLT and subsequently added to each tumor sample. The tissue was disrupted and homogenized using a 20-gauge needle. An equal volume of 70% ethanol was added, transferred to a RNeasy spin column, and centrifuged for 30 s at 12,000 rpm. The flow-through was discarded. Buffer RW1 was added to the RNeasy spin column and centrifuged at 12,000 rpm for 30 s. The flow-through was discarded. Residual DNA was removed using the RNase-Free DNase Set (Qiagen cat. #79254) according to the manufacturer’s protocol. RPE was added to the RNeasy spin column and centrifuged at 12,000 rpm for 30 s. This step was repeated and centrifuged at the same speed for 2 min. RNeasy spin column was placed in a clean 2-ml collection tube. Samples were eluted with 50 ul of RNase-free water for 1 min at 12,000 rpm. Samples were analyzed by the nanodrop to detect concentration and 260/230 ratio. RNA purity was assessed using the Agilent Bioanalyzer 4200 at the Molecular Biology Core Facility of Dana-Farber Cancer Institute.

### Immune profile gene analysis

Purified RNA was isolated from murine tumors. Isolated RNA was submitted to the Center for Advanced Molecular Diagnostics core facility at Brigham and Women’s Hospital. Gene expression analysis was conducted using the nCounter PanCancer Immune Profiling panel which includes 770 immune-related genes and relevant controls. NanoString gene expression values were normalized using the best subset of the 40 reference genes included in the panel, as determined by the geNorm algorithm [60]. The nSolver Advanced Analysis 2.0 software was used to perform all normalization. Pathway signatures were calculated by condensing biologically related groups of genes using the first principal component of their expression data [61]. Cell type scores were calculated using the average log2 normalized expression of each cell type’s marker genes. The cell type abundance scoring is modified from other reports [62] where strict cell type gene correlation-driven QC p values were determined based on data that passed QC.

## Results

### Tumor-infiltrating leukocyte populations are significantly altered between the 4 murine breast cancer models

**Fig. 1:**
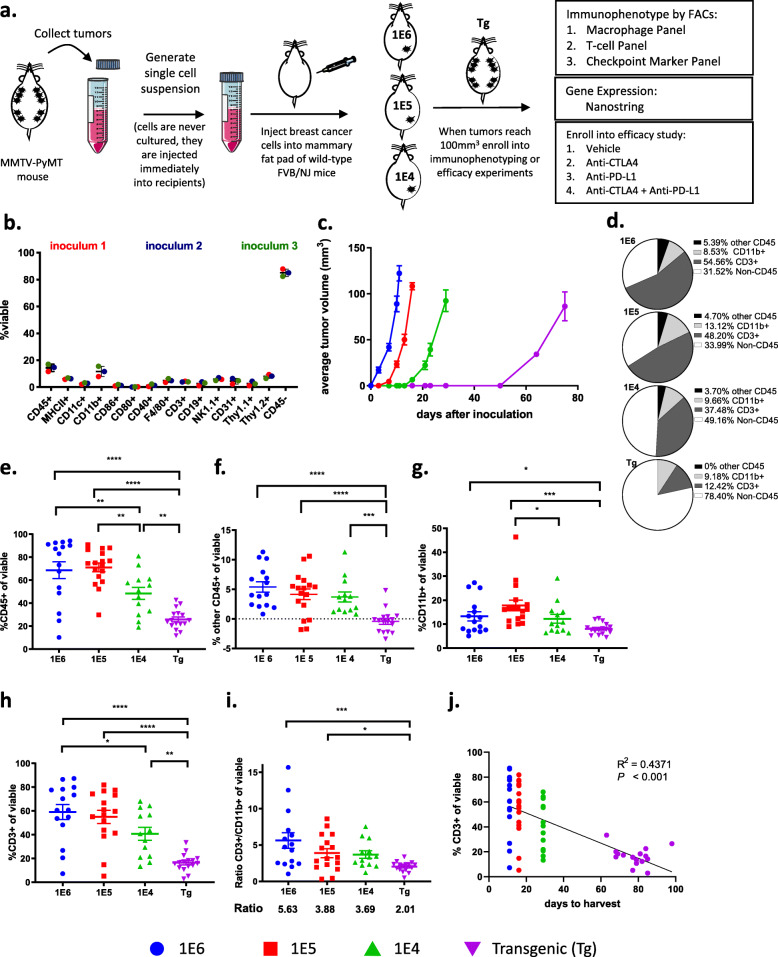
The number of cancer cells inoculated in preclinical models influences tumor growth kinetics as well as the tumor microenvironment. **a** Tumors from the autochthonous MMTV-PyMT model were harvested and single-cell suspensions were generated. Cells (1E6, 1E5, or 1E4) were injected into the mammary fat pad of FVB/NJ wild-type mice. When the tumors reached 100 mm^3^, mice were randomized into an experimental group. **b** Tumor inoculum was generated on three separate days, and flow cytometry was performed to identify the cell proportions. **c** Tumor volumes were measured every 3–4 days until individual tumors reached 100 mm^3^; tumor volumes are plotted as average tumor burden ± SEM. **d**–**j** Tumors were obtained when they reached 100 mm^3^, and flow cytometry was performed. **d** Proportion of major cell types are shown. **e** Percent of CD45^+^ cells out of total viable cells. **f**–**h** Proportion of CD3^+^, CD11b^+^, and other CD45^+^ cells of total viable cells. **i** Ratio of CD3^+^ to CD11b^+^ out of viable cells. **j** Regression analysis of the proportion of CD3^+^ out of viable cells, to time for tumor to reach 100 mm^3^. Graphs show mean ±SEM from at least two independent experiments with at least three mice per group. Each data point represents an individual mouse. *P<0.05, **P<0.01, and ***P<0.001

Three versions of preclinical breast cancer models were generated by isolating tumors from autochthonous MMTV-PyMT mice, dissociating the tumors into single-cell suspensions, referred to as inoculum, and injecting 1E6, 1E5, or 1E4 cells into the 4th mammary fat pad of wild-type FVB/NJ mice (Fig. 1a). Flow cytometry was used to assess the composition of cells in the inoculum that were injected into recipient mice (Supl. Fig. 1a-b), which revealed that the cell suspension was composed of approximately 15% CD45^+^ and 85% CD45^-^ cells (Fig. 1b). The CD45^+^ cells represented myeloid cells (CD11b^+^, CD11c^+^, F4/80^+^), T cells (CD3^+^), B cells (CD19^+^), and NK cells (NK1.1^+^; Supl. Fig. 1b) and the CD45^-^ cells consisted of tumor cells as well as a small percent of both endothelial cells (CD31^+^) and fibroblasts (Thy1.1^+^ and Thy1.2^+^; Fig. 1b). Tumor latency was directly related to the number of cells that were inoculated into the mammary fat pad, as inoculating fewer cells correlated with a longer time to reach 100 mm^3^ (Fig. 1c). It took approximately 10, 15, and 30 days for tumors to reach a volume of 80–100 mm^3^ in the 1E6, 1E5, and 1E4 models, respectively, while it took approximately 80 days for each tumor on the autochthonous model to reach 100 mm^3^ (Fig. 1c).

To test if tumor latency influences immune cell infiltration or phenotype, immunophenotyping was performed on tumors generated in each breast cancer model. For baseline immune profiling, tumors were harvested when they reached 100 mm^3^ because this is generally the size of tumors when animals are enrolled in efficacy studies. In addition, tumors that are 100 mm^3^ allow for immune cell analysis without areas of tumor necrosis. Flow cytometry revealed a significant difference in the frequency of leukocytes (CD45^+^ cells) within the tumor as a percentage of total viable cells (Fig. 1d). Tumors with the shortest latency (1E6 and 1E5 models) had the highest absolute number of infiltrating leukocytes as measured by CD45^+^ cells out of total viable cells (Fig. 1e, Supl. Fig.1c). Strikingly, in the 1E6 and 1E5 models, immune cells were the major cell type within tumors, representing more than 60% of the total cells in the tumor which may represent an acute inflammatory response from the inoculation (Fig. 1e). T cells (CD3^+^) and myeloid cells (CD11b^+^) made up the majority of the CD45^+^ cells; with less than 5% contribution from other CD45^+^ cells (Fig. 1f–h). Myeloid cells (percent of CD11b^+^ cells out of a total of viable cells) were consistent in their frequency between the syngeneic models; however, the autochthonous model had significantly fewer myeloid cells than the 1E6 and 1E5 models (Fig. 1d,g). T cells (percent of CD3^+^ cells out of total viable cells) accounted for a substantial fraction of the CD45^+^ cells in the 1E6 and 1E5 syngeneic models and was less robust in the 1E4 and transgenic models (Fig. 1d,h). This resulted in differences in the ratio of T-cells to myeloid cells, where tumor models with a shorter tumor latency had the highest T cell ratios. The 1E6 model had 5.63 T cells for every 1 myeloid cell whereas the autochthonous model had 2.01 T cells for every 1 myeloid cell (Fig. 1i). Additionally, a correlation was observed between tumor latency and the number of T cells within the tumor (Fig. 1j).

### Subset characterization reveals differences in tumor T cell immune populations among the 4 versions of the MMTV-PyMT breast cancer models

**Fig. 2:**
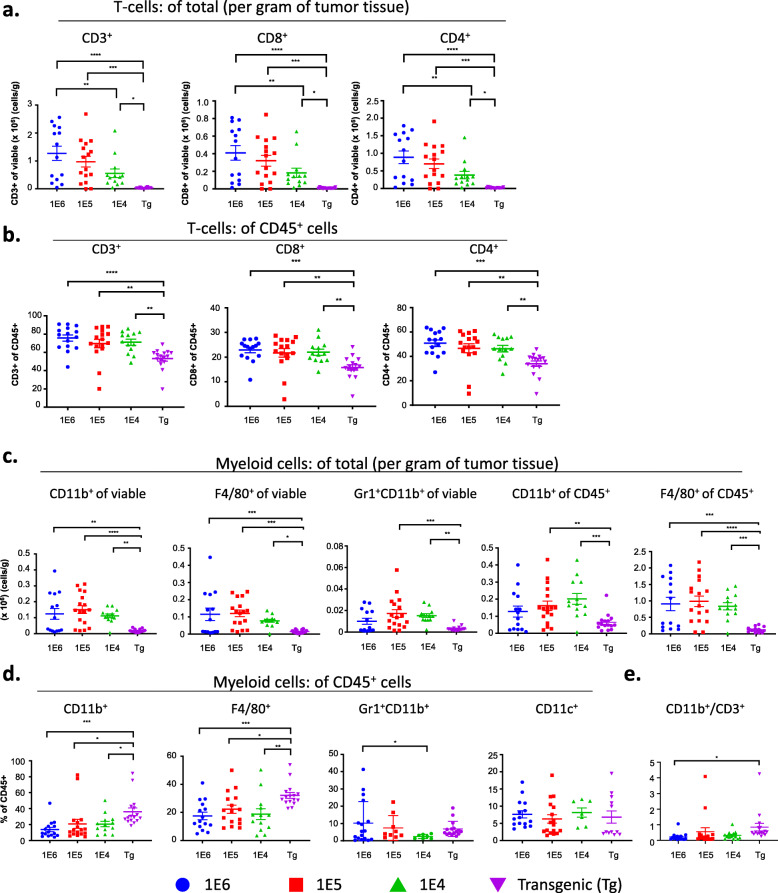
Tumor-infiltrating leukocyte populations differ significantly in the different versions of the MMTV-PyMT breast tumor model. Tumors from the autochthonous MMTV-PyMT model were harvested and single-cell suspensions were generated. Cells (1E6, 1E5, or 1E4) were injected into the mammary fat pad of FVB/NJ wild-type mice. When the tumors reached 100 mm^3^, tumors were obtained and processed into a single-cell suspension for immunophenotyping by flow cytometry. **a** Analysis comparing the absolute number of lymphocytes (CD3^+^) and lymphocyte subsets (CD8^+^ and CD4^+^) per gram of tissue. **b** Analysis comparing the frequency of lymphocytes (CD3^+^) and lymphocyte subsets (CD4^+^ and CD8^+^) out of total CD45^+^ cells. **c** Analysis comparing the absolute number of myeloid cells (CD11b^+^) and myeloid cell subsets (F4/80^+^ and Gr1^+^CD11b^+^) per gram of tissue. **d** Analysis of myeloid cell subsets as a percent of total CD45^+^ cells. Graphs show mean ±SEM from at least two independent experiments with at least three mice per group. Each data point represents an individual mouse. Unpaired two-tailed t test. *P<0.05, **P<0.01, *** *P*<0.001, and **** *P*<0.001

**Fig. 3:**
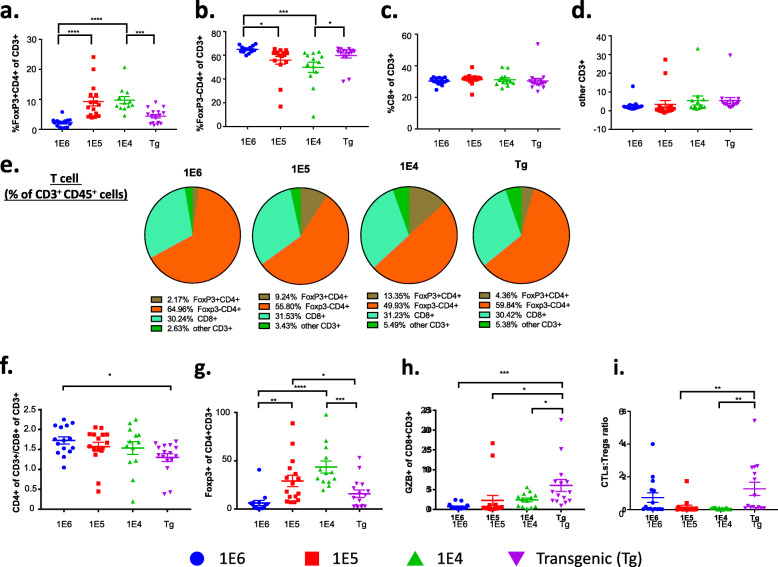
T cell immune subsets differ significantly between the different versions of the MMTV-PyMT breast tumor model. Tumors from the autochthonous MMTV-PyMT model were harvested and single-cell suspensions were generated. Cells (1E6, 1E5, or 1E4) were injected into the mammary fat pad of FVB/NJ wild-type mice. When the tumors reached 100 mm^3^, tumors were obtained and processed into a single-cell suspension for immunophenotyping by flow cytometry. **a**–**d** Analysis of T cell subsets as a percent of CD3^+^ cells and include: **a** T-regulatory cells (FoxP3^+^CD4^+^), **b** FoxP3^-^CD4^+^, **c** CD8^+^, and **d** other CD3^+^ cells. **e** Analysis of lymphocyte cell subsets as a percent of viable cells represented as pie charts. **f** The ratio of CD4^+^ to CD8^+^ out of total lymphocytes. **g** Analysis of T regulatory cells represented by FoxP3^+^ of CD4^+^ cells. **h** The ratio of CTLs represented by GZB^+^ of CD8^+^. **i** The ratio of CTLs to T-regulatory cells. Graphs show mean ± SEM from at least two independent experiments with at least three mice per group. Each data point represents an individual mouse. Unpaired two-tailed t test. *P<0.05, **P<0.01, ***P<0.001, and ****P<0.001

To confirm the absolute numbers of immune cells in tumors, we determined the proportion of T cells per gram of tumor tissue as well as their frequency out of CD45^+^ cells. The extent of T cell infiltration out of gram of tissue (Fig. 2a) was similar to the findings out of total viable cells (Fig. 1h), which reflects the absolute increase in numbers of T cells out of all cells in the tumor. This trend was consistent with the absolute numbers of CD8^+^ and CD4^+^ T cells (Fig. 2a). The difference in absolute numbers of T cells prompted further investigation into immune cell subpopulations and their respective phenotypes. Using flow cytometry, T cell populations were characterized out of CD3^+^ cells including CD4^+^FoxP3^+^ (T regulatory cells; Tregs), CD4^+^FoxP3^-^, and CD8^+^ T cells (gating strategy shown in Supl. Fig. 2a). The absolute number of CD8+ and CD4+ cells in the syngeneic models was significantly higher than the autochthonous model (Fig 2a). The same pattern emerged when gated as a frequency out of CD45^+^ cells (Fig. 2b). Tregs are responsible for dampening the cytotoxic T lymphocyte (CTL) responses and are characterized by expression of the transcription factor FoxP3 [63]. The Treg population increased in the 1E5 and 1E4 models (Fig. 3a,b), and there were no changes in the frequency of CD8 T cells out of total CD3^+^ cells (Fig. 3c,e). The ratio of CD4^+^CD3^+^ cells over CD8^+^CD3^+^ cells was highest in the 1E6 model (Fig. 3f). A different gating strategy was used to identify the frequency of FoxP3^+^ cells out of CD4^+^ T cells (Tregs) as well as the frequency of granzyme b (GrB)^+^ CD8^+^ T cells (cytotoxic T lymphocytes; CTLs). This revealed that the 1E5 and 1E4 models again had the highest frequency of Tregs, whereas the autochthonous model had the highest frequency of GrB^+^CD8^+^ CTLs (Fig. 3g,h). This resulted in a significant increase in the CTL:Treg ratio in the autochthonous model compared to the 1E5 and 1E4 models (Fig. 3i).

### Subset characterization reveals differences in tumor myeloid cell populations among the 4 versions of the MMTV-PyMT breast cancer models

**Fig. 4:**
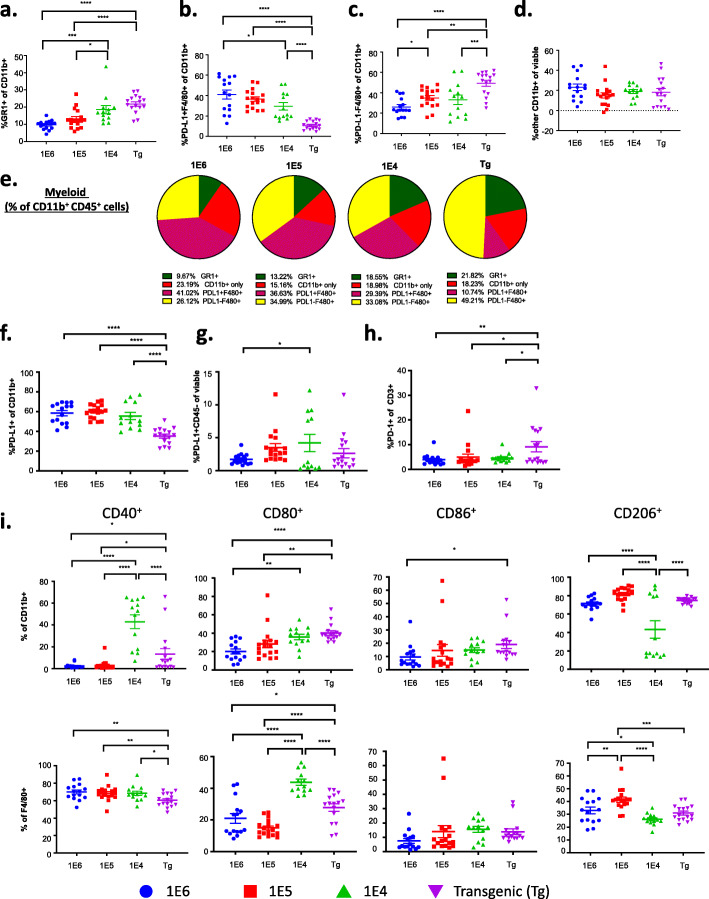
Myeloid immune cell subsets differ significantly between the different versions of the MMTV-PyMT breast tumor model. Tumors from the autochthonous MMTV-PyMT model were harvested and single-cell suspensions were generated. Cells (1E6, 1E5, or 1E4) were injected into the mammary fat pad of FVB/NJ wild-type mice. When tumors reached 100 mm^3^, tumors were obtained and processed into a single-cell suspension for immunophenotyping by flow cytometry. **a**–**d** Analysis of cells subsets as a percent of myeloid cells (CD11b^+^) include **a** myeloid-derived suppressor cells **(**MDSC (GR1^+^)), **b** PD-L1^+^F4/80^+^, **c** PD-L1^–^ F4/80^+^, and **d** other CD11b^+^ populations; **e** Analysis of the myeloid cells subsets as a percent of viable cells include **f** analysis of PD-L1 expression on myeloid cells (CD11b^+^) and **g** cancer cells represented by CD45^-^. **h** Analysis of the ratio of lymphocytes expressing PD-1. **i** Analysis of M1-like macrophage markers, CD40^+^, CD80^+^, and CD86^+^ and an M2-like macrophage marker, CD206^+^, as a ratio of total myeloid cells (CD11b^+^, top), and mature macrophages (F4/80^+^, bottom). Graphs show mean ± SEM from at least two independent experiments with at least three mice per group. Each data point represents an individual mouse. Unpaired two-tailed t test. *P<0.05, **P<0.01, ***P<0.001, and ****P<0.001

Further analysis of myeloid (CD11b^+^) cells revealed significant differences in myeloid subpopulations between the four versions of the MMTV-PyMT breast tumor models (Fig. 4a–e). While the autochthonous model had the fewest number of myeloid cells out of viable cells (Fig. 1g), as well as out of gram of tumor (Fig. 2c), it had the highest frequency of myeloid cells (CD11b^+^) and macrophages (F4/80^+^) out of total CD45^+^ immune cells (Fig. 2d). The other myeloid cell populations (Gr1^+^CD11b^+^ and CD11c^+^) were relatively low and unchanged between the four versions of the MMTV-PyMT breast models, except for a small, yet significant increase in MDSCs (Gr1^+^CD11b^+^) in the 1E6 compared to the 1E4 model (Fig. 2d). There was a 1:1 ratio of CD11b^+^ to CD3^+^ of CD45^+^ cells in the transgenic model, which was the highest ratio among the four models (Fig. 2e). Therefore, while there are fewer immune cells in the TME of MMTV-PyMT tumors compared to the syngeneic tumors, the percent of myeloid cells out of CD45 cells is highest. The autochthonous and 1E4 models had the highest proportion of Gr1^+^ cells out of CD11b^+^ cells (Fig. 4a); this phenotype is generally characterized as myeloid-derived suppressor cells [64, 65]. The F4/80^+^ cells in the autochthonous model were largely PD-L1 negative, in contrast to F4/80^+^ cells in the syngeneic models, which had the highest proportion of PD-L1-positive F4/80 cells (Fig. 4b,c,e). A different gating strategy was used to assess PD-L1^+^ cells out of CD11b^+^ cells as well as of CD45^-^ cells and demonstrated that the frequency of PD-L1 expression on myeloid cells was lowest in the autochthonous model, and there was no change on CD45-negative cells (Fig. 4f,g). Interestingly, the autochthonous model had the highest frequency of PD-1^+^ T cells out of CD3^+^CD45^+^ cells (Fig. 4h).

Our group previously described that using macrophage targeting compounds to convert pro-tumor macrophages to an anti-tumor phenotype induced reduction of primary and metastatic tumors in the MMTV-PyMT autochthonous model, indicating that the myeloid cell population is a major contributor to disease progression [45, 66]. The phenotype of tumor macrophages has previously been shown to correspond to drug sensitivity and disease outcome in this model; therefore, we further investigated macrophage phenotype across the different models [45, 46]. Markers CD40, CD80, and CD86 were used to identify “M1”-like macrophages and CD206 was used as a marker of “M2”-like macrophages. The autochthonous model and the 1E4 model had the highest frequency of M1-like myeloid and macrophage populations (Fig. 4i and Supl. Fig. 3a–c) suggesting that the 1E6 and 1E5 models had more suppressive myeloid cells, in line with their higher frequencies of PD-L1^+^ myeloid cells (Fig. 4f).

### Immunophenotyping reveals differences in the tumor microenvironment in two versions of the EMT6 syngeneic breast cancer model

The EMT6 model of TNBC was employed as a second model to test how tumor cell number and latency influenced the resultant TME. Mice were injected with either 1E6 or 1E4 tumor cells, and when tumors reached 100–150 mm^3^, tumors were isolated for flow cytometry (Supl. Fig. 4a). The time to reach 100 mm^3^ differed in the two versions of the EMT6 models, where 1E6 and 1E4 tumors took 4 and 11 days, respectively (Supl. 4a). Using flow cytometry differences in the TME were then evaluated. Indeed, as seen in the MMTV-PyMT models, 1E6 tumors had more T cells (28% out of CD45^+^) and fewer myeloid cells (18.9% out of CD45^+^) whereas the 1E4 tumors had almost no T cells (5.9% out of CD45^+^), but were highly infiltrated with myeloid cells (85.5% out of CD45^+^) (Supl. Fig. 4b-e). These findings are consistent with the observations from the MMTV-PyMT model, suggesting these observations extend beyond the MMTV-PyMT syngeneic models.

### The 4 versions of the MMTV-PyMT breast cancer model have distinct tumor immune transcriptional profiles

**Fig. 5:**
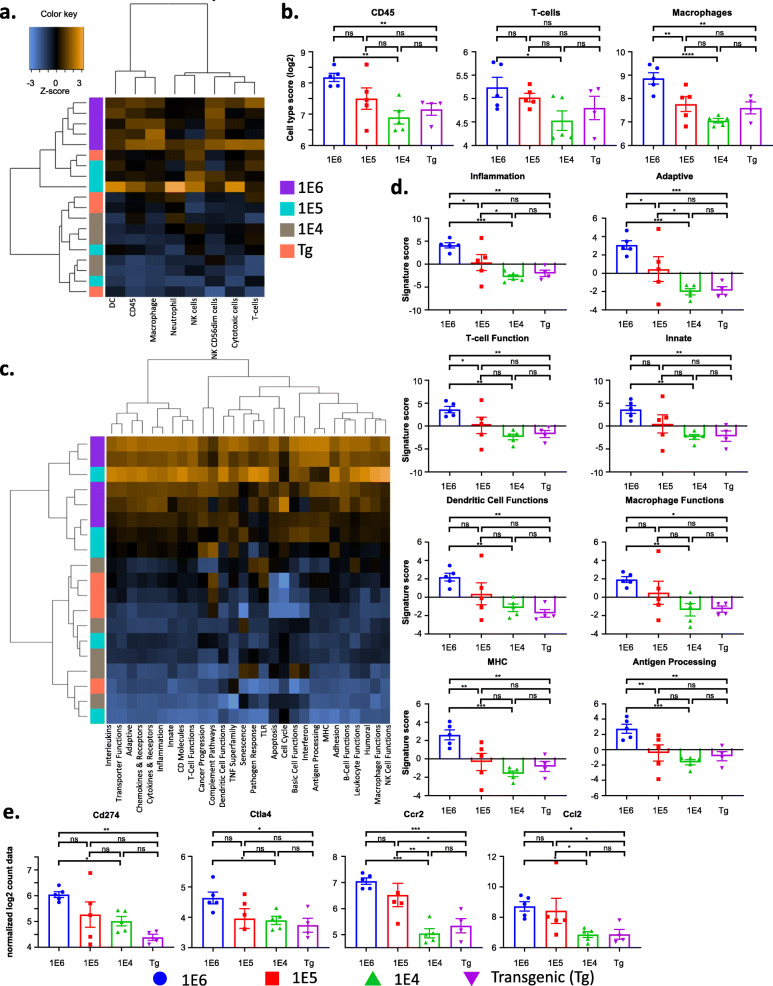
The different versions of the MMTV-PyMT breast tumor model have distinct immune transcriptional signatures. Tumors from the autochthonous MMTV-PyMT model were harvested and single-cell suspensions were generated. Cells (1E6, 1E5, or 1E4) were injected into the mammary fat pad of FVB/NJ wild-type mice. When tumors reached 100 mm^3^, tumors were obtained, and RNA was extracted from tumors to assess transcriptional changes. The NanoString PanCancer Immune Profiling Panel was used to analyze gene expression patterns related to the immune cell compartment of the tumors. **a** A heatmap of all cell-type scores is shown. **b** Quantification of cell types scores of immune cells, T cells, and macrophages are shown. **c** Hierarchically clustered heatmap showing the pathway scores across the four models. Yellow and blue represent upregulated and downregulated scores, respectively. **d** Signature scores from the following pathways are highlighted: inflammation, adaptive immunity, T cell function, innate immunity, dendritic cell functions, macrophage functions, MHC, and antigen processing. **e** Normalized log_2_ counts are shown for the following transcripts: Cd274 (PD-L1), Ctla4, Ccr2, and Ccl2. Graphs show mean ± standard deviation with 4–5 mice per group. Each data point represents an individual mouse. Unpaired two-tailed t test. *P<0.05, **P<0.01, ***P<0.001, and ****P<0.001

To interrogate mRNA transcripts expressed by cells in each of the four versions of the MMTV-PyMT breast tumors, transcriptional profiling was performed by NanoString analysis (Supl. Fig. 5-6). Since the analysis was performed on bulk tumor tissue, the data would correspond most similarly to flow cytometry analysis of total viable cells and per gram of tumor tissue. The autochthonous tumors clustered most closely with the 1E4 tumors, while the 1E6 and 1E5 tumors are clustered together (Fig. 5 and Supl. Fig. 5d). Cell type score analysis validated the flow cytometry data, where the 1E6 model had the highest scores for all immune cell types profiled, including T cells and macrophages (Fig. 5a–c and Supl. Fig. 5e). In addition, there was no change in neutrophils, but the 1E6 and 1E5 model had the highest scores for dendritic cells, and the 1E6 model had the highest score for activated natural killer cells (NK CD56dim; Supl. Fig. 5f).

Unsupervised hierarchical clustering showed 1E6 and 1E5 models clustered most similarly and the 1E4 and autochthonous models clustered most similarly on activated pathway scores (Fig. 5c). Pathway analysis further revealed increased scores for immune-related pathways including both adaptive and innate immunity, and macrophage and T-cell function. In general, the 1E6 and 1E5 models had the highest scores for all immune-related pathways (Fig. 5d and Supl. Fig. 6, Tables 1, 2 and 3), likely due to the increased number of immune cells in the tumors that were identified by flow cytometry. The most striking differences were observed when comparing the autochthonous model with the 1E6 model, where multiple transcripts associated with antigen presentation and immune signaling were increased in the 1E6 model (Supl Fig. 7a-f). More specifically, the 1E6 model had the highest levels of Cd274 (PD-L1), Ctla4, Ccr2, and Ccl2 (Fig. 5e).

**Table 1:** List of Top 20 differential expression of immune transcripts in 1E6 versus the autochthonous model

Transcript	Gene Sets	Probe ID	Log2 fold change	*P*-value
Tfrc-mRNA	CD molecules, Transporter Functions	NM_011638.3:1930	1.52	9.64E-09
Birc5-mRNA	Apoptosis, Cell Cycle, Cytokines & Receptors	NM_009689.2:237	1.68	3.66E-08
Psma2-mRNA	Cancer Progression	NM_008944.2:136	0.26	9.74E-07
Runx1-mRNA		NM_001111021.1:3055	-1.13	1.38E-06
Il1rap-mRNA	Cytokines & Receptors, Inflammation, Innate, Interleukins	NM_008364.2:2415	0.96	1.70E-06
Hif1a-mRNA	Apoptosis, Cancer Progression	NM_010431.2:1294	0.53	1.94E-06
Nt5e-mRNA	B-Cell Functions, CD molecules, Inflammation	NM_011851.3:1600	1.54	5.90E-06
Oas2-mRNA	Basic Cell Functions	NM_145227.3:414	4.07	7.48E-06
Fcgr2b-mRNA	Antigen Processing, B-Cell Functions, CD molecules, Inflammation, Interleukins, MHC, Transporter Functions	NM_001077189.1:1225	2.38	7.55E-06
Ltbr-mRNA	Apoptosis, TNF Superfamily	NM_010736.3:1962	0.27	8.11E-06
Saa1-mRNA	Adhesion, Cytokines & Receptors, Innate, Macrophage Functions	NM_009117.3:351	-2.47	8.41E-06
Smn1-mRNA	Cancer Progression	NM_011420.2:390	0.24	9.88E-06
Itgb4-mRNA	Adhesion, CD molecules	NM_001005608.2:3355	1.26	1.04E-05
Bax-mRNA	Apoptosis, Transporter Functions	NM_007527.3:735	0.57	1.24E-05
Bst2-mRNA	CD molecules, Humoral, Innate	NM_198095.2:468	2.4	1.28E-05
Igf2r-mRNA	Apoptosis, CD molecules, Transporter Functions	NM_010515.1:2585	0.72	1.30E-05
Traf3-mRNA	Apoptosis, Cytokines & Receptors, Innate, TLR	NM_001048206.1:6385	0.53	2.45E-05
Ikzf2-mRNA	T cell Functions	NM_011770.4:7230	1.22	2.52E-05
Ddx58-mRNA	Innate, Interferon	NM_172689.3:1751	1.32	3.00E-05
Ccl27a-mRNA	Cytokines & Receptors	NM_001048179.1:265	-0.79	3.15E-05

**Table 2:** List of Top 20 differential expression of immune transcripts in 1E5 versus autochthonous model

Transcript	Gene Sets	Probe ID	Log2 fold change	*P*-value
Birc5-mRNA	Apoptosis, Cell Cycle, Cytokines & Receptors	NM_009689.2:237	1.67	3.78E-08
Hif1a-mRNA	Apoptosis, Cancer Progression	NM_010431.2:1294	0.653	7.39E-08
Runx1-mRNA		NM_001111021.1:3055	-1.36	1.32E-07
Psma2-mRNA	Cancer Progression	NM_008944.2:136	0.22	4.69E-07
Ltbr-mRNA	Apoptosis, TNF Superfamily	NM_010736.3:1962	0.30	2.77E-06
Tfe3-mRNA	Humoral	NM_172472.3:2715	-0.56	3.63E-06
Tfrc-mRNA	CD molecules, Transporter Functions	NM_011638.3:1930	0.91	6.57E-06
Saa1-mRNA	Adhesion, Cytokines & Receptors, Innate, Macrophage Functions	NM_009117.3:351	-2.45	8.98E-06
Bax1-mRNA	Apoptosis, Transporter Functions	NM_007527.3:735	0.59	9.66E-06
Smad4-mRNA	Cancer Progression	NM_008540.2:2885	-0.31	1.88E-05
Ccl27a-mRNA	Cytokines & Receptors	NM_001048179.1:265	-0.77	2.64E-05
Dusp4-mRNA	Basic Cell Functions, Innate	NM_176933.4:2200	1.29	3.53E-05
Mapk14-mRNA	Innate, Senescence, Transporter Functions	NM_001168513.1:114	0.25	4.33E-05
Glycam1-mRNA	Adhesion	NM_008134.2:124	-5.41	4.39E-05
Casp1-mRNA	Cytokines & Receptors, Innate, Interleukins, Microglial Functions	NM_009807.2:259	1.01	5.58E-05
Tank-mRNA	Basic Cell Functions, Innate	NM_011529.1:491	0.33	8.05E-05
Ctsl-mRNA	Basic Cell Functions	NM_009984.3:45	-0.69	8.31E-05
Jak3-mRNA	B-Cell Functions, Cytokines & Receptors, Innate, Interleukins, T-Cell Functions	NM_010589.5:145	-0.71	8.42E-05
Ikzf1-mRNA	B-Cell Functions, NK Cell Functions, T-Cell Functions	NM_001025597.1:4420	-1.66	1.22E-04
Psmb7-mRNA	Basic Cell Functions	NM_011187.1:184	0.22	1.65E-04

**Table 3:** List of Top 20 differential expression of immune transcripts in 1E4 versus autochthonous model

Transcript	Gene Sets	Probe ID	Log2 fold change	*P*-value
Glycam1-mRNA	Adhesion	NM_008134.2:124	-9.09	1.34E-07
Itgb4-mRNA	Adhesion, CD molecules	NM_001005608.2:3355	1.73	2.23E-07
Birc5-mRNA	Apoptosis, Cell Cycle, Cytokines & Receptors	NM_009689.2:237	1.43	2.83E-07
Saa1-mRNA	Adhesion, Cytokines & Receptors, Innate, Macrophage Functions	NM_009117.3:351	-3.13	5.19E-07
Il6ra-mRNA	CD molecules, Chemokines & Receptors, Cytokines & Receptors, Interleukins	NM_010559.2:2825	-1.61	5.86E-07
Hif1a-mRNA	Apoptosis, Cancer Progression	NM_010431.2:1294	0.53	1.12E-06
Tnfrsf10b-mRNA	Apoptosis, CD molecules, TNF Superfamily	NM_020275.3:1625	0.93	1.40E-06
C4b-mRNA	Complement Pathway, Humoral, Inflammation, Innate	NM_009780.2:491	-2.51	6.68E-06
Il1rap-mRNA	Cytokines & Receptors, Inflammation, Innate, Interleukins	NM_008364.2:2415	0.84	7.95E-06
Cd200-mRNA	CD molecules	NM_010818.3:686	1.19	8.63E-06
Casp1-mRNA	Cytokines & Receptors, Innate, Interleukins, Microglial Functions	NM_009807.2:259	1.13	1.76E-05
Gpi1-mRNA	Apoptosis, Cytokines & Receptors, Humoral	NM_008155.4:1540	0.53	2.43E-05
Mif-mRNA	B-Cell Functions, Cytokines & Receptors, Inflammation, Innate, Transporter Functions	NM_010798.2:373	0.62	2.84E-05
Vegfa-mRNA	Apoptosis, Cytokines & Receptors, Macrophage Functions, T-Cell Functions	NM_001025250.3:3015	1.78	2.85E-05
Kit-mRNA	CD molecules, Cytokines & Receptors	NM_001122733.1:4275	1.06	3.52E-05
Tank-mRNA	Basic Cell Functions, Innate	NM_011529.1:491	0.35	4.55E-05
Lcp1-mRNA	T-Cell Functions, Transporter Functions	NM_001247984.1:3344	-0.59	5.19E-05
Bax-mRNA	Apoptosis, Transporter Functions	NM_007527.3:735	0.49	6.44E-05
Atm-mRNA	Apoptosis, B-Cell Functions, Cell Cycle, Senescence	NM_007499.2:5543	-0.62	7.50E-05
App-mRNA	Apoptosis, Cell Cycle, Innate, Transporter Functions	NM_007471.2:511	0.64	8.04E-05

### The four versions of the MMTV-PyMT breast cancer model respond differently to immune checkpoint blockade

**Fig. 6:**
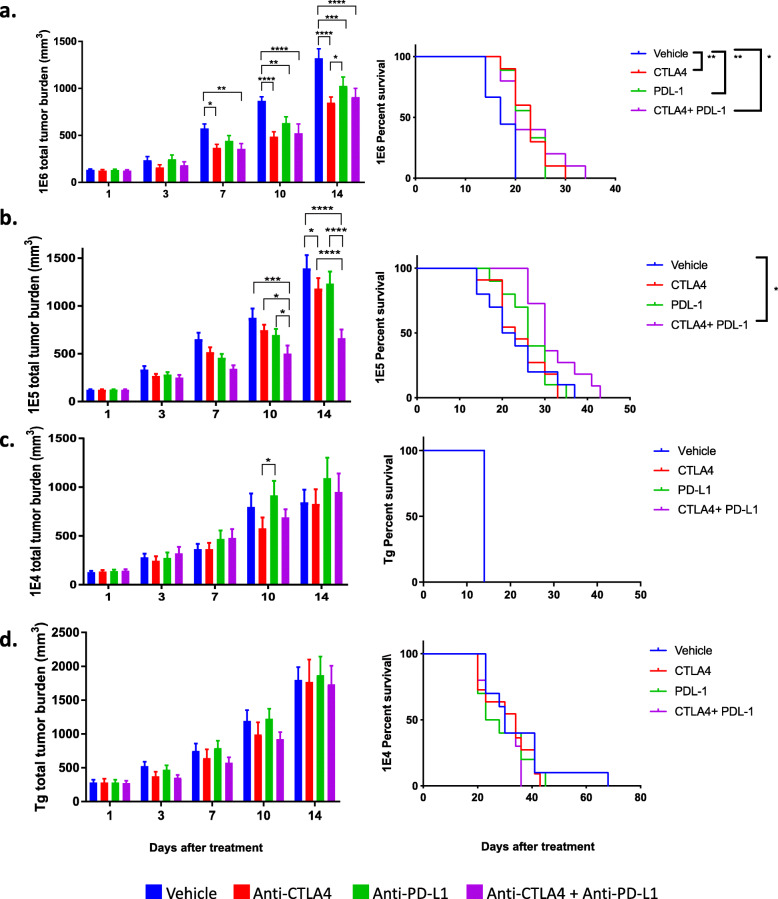
The different versions of the MMTV-PyMT syngeneic model respond differently to immune checkpoint blockade. Tumors from the autochthonous MMTV-PyMT model were harvested and single-cell suspensions were generated. Cells (1E6, 1E5, or 1E4) were injected into the mammary fat pad of FVB/NJ wild-type mice. When the tumors reached 100 mm^3^, mice were randomized into four treatment groups: vehicle (IgG2B), anti-PD-L1, anti-CTLA-4, and the combination of anti-PD-L1 and anti-CTLA-4. Tumor volumes were measured and plotted as the mean total tumor burden ± SEM (left) and survival analysis of mice are shown (right). Shown is a representative experiment of two individual experiments n=3–7/treatment groups for the 1E6 (**a**), 1E5 (**b**), 1E4 (**c**), and transgenic models (**d**). In Fig. 6d, all mice were sacrificed on the same day due to meeting endpoint criteria. Two-way ANOVA multiple comparisons and Gehan-Breslow-Wilcoxon tests were performed. *P<0.05, **P<0.01, ***P<0.001, and ****P<0.001

We and others have previously shown that the MMTV-PyMT murine model of breast cancer is resistant to checkpoint blockade as a monotherapy [45, 67]. We show here that the MMTV-PyMT model had the least number of infiltrating leukocytes and lymphocytes within the TME compared to the 3 syngeneic models. Since the syngeneic models had a higher absolute number of T cells, as well as a higher proportion of T cells expressing PD-1 and myeloid cells that express PD-L1, we hypothesized that the syngeneic models would demonstrate enhanced responses to ICB. Indeed, the 1E6 model, which had the highest frequency of T cells and PD-L1^+^ myeloid cells, was the only model among the four that responded to anti-PD-L1 and anti-CTLA-4 as monotherapy after 14 days of treatment as seen by delayed tumor progression (Fig. 6, Supl. Fig. 8). In the 1E6 model, the combination of anti-PD-L1 plus anti-CTLA-4 was significant over vehicle control but not over either monotherapy. This resulted in a small yet significant increase in overall survival (Fig. 6a). The 1E5 model had a small yet significant decrease in total tumor burden with the anti-CTLA-4 monotherapy at day 14, but did not translate to an increase in overall survival; however, the combination of anti-CTLA-4 plus anti-PD-L1 induced a reduction of tumor burden at 14 days that led to improved overall survival (Fig. 6b, Supl. Fig. 7b). Both the 1E4 and the autochthonous model were resistant to monotherapy as well as combination therapy (Fig. 6c-d, Supl. Fig. 8c-d).

**Fig. 7:**
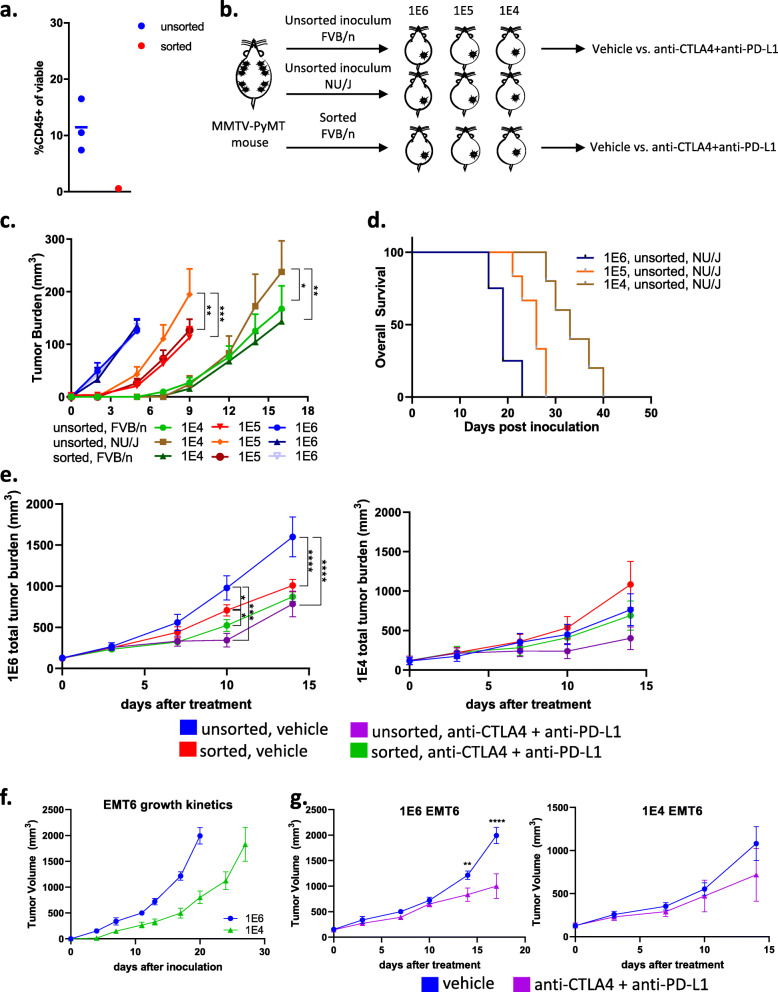
Testing tumor growth kinetics and immunotherapy response in immunodeficient and EMT6 murine models. Tumors from the autochthonous MMTV-PyMT model were harvested and single-cell suspensions were generated. **a** CD45+ cells were successfully removed (“sorted”). **b** Cells (1E6, 1E5, or 1E4) were injected into the mammary fat pad of FVB/NJ wild-type or nude mice. **c-d** Tumor volumes were measured and plotted as the mean total tumor burden ± SEM and the survival of nude mice are shown (**d**). **e** When the tumors reached 100 mm^3^, mice were randomized to treatment with Vehicle (IgG2B) or the combination of anti-PD-L1 plus anti-CTLA-4. **f**–**g** Babl/c mice were implanted with 1E6 or 1E4 tumor cells, and tumor growth kinetics (**f**) and response to vehicle and combination of anti-PD-L1 plus anti-CTLA-4 (**g**) were plotted. Tumor volumes were measured and plotted as the mean total tumor burden ± SEM. *P<0.05, **P<0.01, ***P<0.001, and ****P<0.001

To test if initial cell density is a driver of response to ICB or if residual immune cells in the inoculum account for these differences, CD45^+^ cells were successfully removed from the inoculum (referred to as sorted) and compared to the original inoculum for their growth rate and response to ICB (Fig. 7a–b). In the immunocompetent FVB/NJ mice all three models (1E6, 1E5, and 1E4) inoculated with unsorted and sorted inoculum grew at similar rates (Fig. 7c). In nude mice, which lack a functional immune system, the unsorted cells grew faster in nude mice compared to FVB/NJ mice, as expected (Fig. 7c). The nude mice reached the endpoint in a cell density-dependent manner (Fig. 7d). In the FVB/n model, when tumors in the 1E6 and 1E4 versions of the model tumors reached 100–150 mm^3^, mice were enrolled into efficacy experiments. FVB/n mice bearing either unsorted (original) or sorted inoculum in the 1E6 or 1E4 versions of the models were treated with either vehicle or anti-CTLA4 plus anti-PD-L1. The unsorted 1E6 inoculum responded to ICB but the 1E4 did not (Fig. 7e), as previously demonstrated (Fig. 6a). The sorted inoculum in the 1E6 model displayed modest yet significant responses to ICB at day 10 but those responses did not continue to day 14 (Fig. 7e). The fact that both the sorted and unsorted 1E6 tumors displayed some responses, while 1E4 sorted and unsorted tumors did not suggest that while immune cells co-injected with tumor cells may play, in part, some role in activating an acute immune response that sensitizes the tumor to ICB, the number of tumor cells used is also important. To further test if different numbers of pure cell cultures would evoke differences in tumor latency, TME and response to ICB, 1E6, and 1E4 EMT6 tumor cells were inoculated into immunocompetent wild-type mice. As with the 1E6 MMTV-PyMT syngeneic tumors, the 1E6 EMT6 tumors demonstrated faster tumor growth compared to corresponding 1E4 tumors (Fig. 7f). When EMT6 tumors reached 100–150 mm^3^, mice were enrolled in efficacy experiments. Similar to the 1E6 MMTV-PyMT syngeneic tumors, the 1E6 EMT tumors had a delay in progression in response to ICB, whereas the 1E4 models were not responsive (Fig. 7g). Taken together these results indicate that tumor cell numbers used to generate tumors correspond to tumor growth kinetics and response to ICB in two different murine models.

To gain insight into what may correlate with ICB response, MMTV-PyMT tumors were regrouped into either predicted responders (1E6 and 1E5 tumors) or predicted non-responders (1E4 and autochthonous; Supl. Fig. 9a). Nanostring gene expression from predicted responders and non-responders clustered by immune cell score, where predicted responders had higher scores related to CD45, T cells, cytotoxic T cells, NK CD56 dim cells, macrophages, and dendritic cells (DCs; Supl. Fig. 9b-c). Responders and non-responders also clustered in the pathway analysis, where the inflammation pathway revealed significant differences between the responders and non-responders (Supl. Fig. 9d-e). This data suggests that total immune cell infiltrate as well exhaustive immune cell phenotypes may correspond to the success of ICB therapy within the context of these versions of the MMTV-PyMT breast cancer model (Supl. Fig. 10).

## Discussion

Mouse models are critical to the rapid and successful translation of preclinical findings to the clinic yet are currently lacking. In addition, there is a critical need for biomarkers to predict response to ICB in breast cancer. Given the substantial heterogeneity of the TME, conclusions based on specific mouse models might limit generalizations, especially regarding the detailed characterization of molecular signaling mechanisms. The MMTV-PyMT autochthonous model has been extensively characterized and is one of the few models available to study Luminal B breast cancer. MMTV-PyMT mice develop spontaneous mammary tumors that closely resemble the progression and morphology of human breast cancers [43, 54, 55]. Notably, gene expression profiling has revealed that MMTV-PyMT tumors cluster closely with ERα-negative “luminal” human breast cancers [68], which is a gene signature similar to the luminal-AR (LAR) TNBC subtype characterized by high AR expression [69] and the molecular apocrine ER/PR-negative, but AR+ tumors described prior to molecular subtyping [70]. The pathology of the autochthonous murine breast tumors provides numerous ways to model human breast cancer in vivo. Here, we report that the 1E6 and 1E5 corresponding syngeneic models do not recapitulate the autochthonous model. Our findings presented here are essential for future preclinical studies and translation to the clinic.

Here, we exploited the MMTV-PyMT and EMT6 syngeneic mouse models to make two major findings. First, the initiating conditions of the tumor (in this case, the number of cells in the inoculum) can dramatically alter the tumor immune microenvironment. Second, we found that these differences in the TME were closely related to the quality of ICB responses (Supl. Fig. 10). We used cells derived from tumors that spontaneously arise in the MMTV-PyMT murine model of breast cancer to generate three versions of the MMTV-PyMT syngeneic models using 1E6, 1E5, or 1E4 cells injected into the mammary fat pad of wild-type FVB/NJ mice. As a second model, we used 1E6 or 1E4 EMT6 cells to generate two versions of the EMT6 tumor model. Our findings are the first to report a detailed characterization of the difference in the TME as a variable of the number of cells injected to generate syngeneic tumors. Importantly, we find that while the 1E6 and 1E5 models responded to ICB, the 1E4 and MMTV-PyMT autochthonous models are resistant. These findings were generalized to the 1E6 and 1E4 EMT6 models as well. The ICB-sensitive tumors demonstrated that protection from the inhibitory effects of Tregs and the presence of high numbers of T cells and macrophages paired with enhanced antigen processing capabilities correlated with response to ICB. These data support our hypothesis that in addition to T cells, M1 macrophages, and other myeloid cells may be required to play a critical role in initiating an anti-tumor immune response. In the clinic, tumors with these characteristics may have greater therapeutic responses to ICB.

The 1E6 and 1E5 MMTV-PyMT tumors had the highest absolute number of T cells. T cells have been used as a prognostic biomarker, yet in this case, infiltration of T cells is likely a response to an acute inflammatory response and not related to T cell recruitment in human tumors. Regardless, the response to ICB correlated with increased T cells (Fig. 3a). Interestingly, there was no correlation between ICB-sensitivity and frequency of CTL numbers or proportions, as the autochthonous model had the highest frequency but was resistant to ICB. The functional activity of T cells depends largely on the expression of co-stimulatory molecules, peptide-MHC complexes, MHC class I molecules, and expression of checkpoint markers (PD-1 and CTLA4) [71]. T cells secrete cytokines to promote a differential effector function. Activated T cells (Th1 type) can secrete IL-2, TNFα, and IFNγ, which in turn induce cytotoxic function of CD8+ T cells and promote phagocytosis through co-stimulatory markers on macrophages and other antigen-presenting cells (CD40, CD86, and CD80) [72, 73]. In contrast, secretion of IL-4, IL-6, IL-10, and IL-13 by Th2 CD4^+^ T cells can promote T cell energy and inhibit the activation of CTLs [46]. We did however see functional differences between responders and the non-responders model in terms of chemokine receptors, cytokines, and interferon and TNF superfamily signatures (Supl. Fig. 6). Tregs correlate with poor prognosis in a variety of epithelial tumor types possibly as a result of dampening T cell immunity in response to cancer-associated antigens [74, 75]. Here, Tregs did not appear to correlate with ICB responses. Another factor worth considering is the low numbers of myeloid cells to facilitate antigen presentation in the autochthonous model, compared to the 1E6 model, which may render the CTLs ineffective in mediating the response to ICB in the autochthonous model. The antigen presentation signature and corresponding genes were significantly increased in the ICB-sensitive models (1E6 and 1E5) compared to the ICB-resistant models (1E4 and autochthonous). This may reflect the fact that the ICB-sensitive tumors received a higher density of cells, and therefore, a potentially higher antigen load was delivered to mice; or it may represent that more CD45+ cells (absolute number) are injected into mice with the inoculum at 1E6. When CD45 cells are removed from the inoculum prior to injection into wild-type FVB/N mice at a density of 1E6, the tumors are no longer sensitive to ICB (Fig. 7e), which may indicate that the residual immune cells injected into the mice in the inoculum activate an immune response and facilitated the recruitment of host T cells. Therefore, increased TILs and antigen presentation may be falsely increased in the sensitive models and may not represent naturally occurring tumors.

An important question, that is currently unknown is if the absolute number of myeloid cells within the TME or the proportion of myeloid cells of total CD45^+^ immune cells is a more important factor for ICB efficacy. The data here suggest that the former is a stronger predictor of response and that the phenotype might not be as critical since the ratio of M1:M2 macrophages was higher in the ICB-resistance models (albeit lower absolute numbers of myeloid cells). Further work to understand TAM phenotype should be carefully noted by their function, signaling pathways, and expression of extracellular markers. We found that the 1E6 and 1E5 models had the highest absolute number of CD11b^+^ myeloid and F480^+^ macrophages, yet the differences were not as pronounced as the difference in T cells. Macrophages play an essential role in T cell activation by presenting antigen and providing activating and stimulatory cytokines essential for T cell function [71]. In addition, macrophages can mediate antibody-dependent cellular toxicity of cancer cells [76] as well as eliminate cancer cells through FcγR-mediated phagocytosis [77]. However, TAMs can also dampen effector T cell function by producing IL-10 that in turn increase their own PD-L1 expression and suppresses cytotoxic T cell responses [78]. The myeloid cells in the 1E6 and 1E5 tumors were more slightly more suppressive than those found in the 1E4 and autochthonous tumor models; indicated by a higher proportion of myeloid cells expressing PD-L1^+^ (Fig. 4f), as well as a lower ratio of M1:M2 macrophages that suggested more M2-like macrophages (Supl. Fig. 3). In line with these observations, we found that transcript levels related to Ccl2 and its receptor were higher in the ICB-sensitive models. CCL2 is a cytokine largely known for its involvement in the recruitment of CCR2+ monocytes from the bone marrow to other sites in the body where they differentiate into macrophages [79, 80]. Additionally, CCL2 has been shown to recruit monocytes and macrophages to breast tumors and to facilitate breast cancer metastasis [81, 82]. The CCL2/CCR2 axis may represent a unique opportunity for anti-cancer therapy and work in this area is already being explored [83, 84]. The combination of CCL2 antagonism with anti-PD-1 has demonstrated efficacy in some mouse models [85]. Taken together, the differences we found in the myeloid compartment was not as striking as those observed for T cells, and importantly, studies have revealed similar outcome for myeloid-targeting strategies between these syngeneic and autochthonous models, where they appear to be able to be used interchangeably [56, 86]. This suggests that myeloid-based immunotherapy studies, but not T cell ICB studies may be suitable in the 1E6 model, but was not directly tested here.

Other studies have shown that the inoculated cell density of 4T1 cells is a determinant of the growth dynamics and metastatic potential of the cells, where injecting fewer cells resulted in extending the time of tumor development to result in 100% metastasis to study metastatic tumors [87]. Table 4 summarizes the use of syngeneic models of breast cancer (4T1, EMT6, and MMTV-PyMT) reported to evaluate PD-(L)1 and CTLA4 blockade efficacy. We observe a lack of standardization of the number of cells inoculated (ranging 5E4-5E6), as well as days after inoculation (7–24 days) and tumor size (40–400 mm^3^), reported at the start of treatment. In an effort to best represent the human disease, the use of mouse models that most closely resemble the human disease is critical. Our study presented here demonstrates that the number of cells injected largely dictates the TME at the start of treatment (100 mm^3^) and response to ICB and should be carefully considered when selecting a model for preclinical studies.

**Table 4:** Published studies on immunotherapy response in syngeneic murine models

Reference	Model	Tumor cells Inoculated	Tumor size (mm^3^)	Time to start treating (d)	Outcome
Kim et al. *PNAS* 2014	4T1	5.00E6	400	11	Tumor eradication with PD-1/CTLA-4 at day 15
	CT26	5.00E6	400	11	Tumor eradication with PD-1/CTLA-4 at day 15
Lian et al Sci Rep 2019	4T1	1.00E5	Not reported	24	PD-L1/CD74 dual blockade reduced lung metastasis
Clift et al. *Cancer Res* 2019	4T1/EMT6	1.00E5	100-150	Not reported	PD-L1 blockade +PVHA inhibited tumor growth
Sun et al. *Mol Cancer Ther* 2020	4T1	1.00E6	100-150	3	CTLA-4 and PD-1 blockade promoted T cell infiltration
Xie et al. *J Immunother Cancer* 2018	4T1	5.00E6	<200	5,8	AngII blockade enhances sensitivity to CTLA-4/PD-1 treatment.
Xu et al. *Clin Cancer Res* 2017	EMT6	5.00E6	Not reported	Not reported	NHS-muIL12 and avelumab combination therapy enhanced antitumor efficacy relative to either monotherapy
Knudson et al. *Oncoimmunology* 2018	EMT6	2.50E5	50-100	Not reported	Bifunctional checkpoint inhibitor of TGFβRII linked to the C-terminus of human anti-PD-L1 heavy chain reduced tumor burden.
	4T1	5.00E4	50-100	Not reported	
Zippelius et al. *Cancer Immunol Res* 2015	EMT6	2.50E5	Not reported	16	PD-L1 overexpression mediates acquired resistance to agonistic anti-CD40 treatment.
	MC38	1.00E6	Not reported	16	
Lewis et al. *Oncoimmunology* 2017	EMT6	1.50E6	Not reported	7	IL-21 inhibition with CTLA4 blockade promoted tumor regression compared to monotherapy.
*Li* et al. Cancer Cell 2018	4T1	5.00E4	<200	6	A monoclonal antibody targeting glycosylated PD-L1 (gPD-L1) blocks PD-L1/PD-1 interaction and promotes PD-L1 internalization and degradation.
	EMT6	5.00E4	<200	6	
Liu et al. *Cancer Discovery* 2016	4T1.2	2.00E4	40-80	16	Neo-adjuvant PD-1/CD-137 therapy had better efficacy than adjuvant
	4T1.2	5.00E4	40-80	10	Increase in tumor-specific CD8+ T cells after neoadjuvant anti-PD-1+anti-CD137 therapy
	E0771	5.00E4	40-80	16-18	Neo-adjuvant PD-1+CD-137 therapy had better efficacy than adjuvant.
Liu et al. *Oncoimmunology* 2019	E0771	2.00E5	50	10	Guadecitabine was similarly effective in the E0771 model of murine breast carcinoma. Finally, we found that guadecitabine in combination with AIT resulted in prolonged survival in both 4 T1 and E0771 breast cancer models.
	4T1	5.00E4	50	10	
Kasikara et al. *Cancer Res* 2019	E0771	1.00E5	Not reported	10	Combination of TAM inhibitor (BMS-777607) and anti-PD-1 improved tumor efficacy by altering the TME.
Messenheimer et al. *Clin Cancer Res* 2017	MMTV-PyMT	1.00E6	<50	7	Sequential combination of anti-OX40 and anti-PD-1 increased efficacy.
Nolan et al. *Sci Transl Med* 2017	MMTV-cre/Brca^1fl/fl^ /p53^+/-^	4.00E4	100	21	Cisplatin treatment combined with dual anti–PD-1 and anti-CTLA-4 therapy substantially augmented antitumor immunity in Brca1-deficient mice.
Young et al. *Plos One* 2016	MMTV-PyMT	1.00E6	Not reported	14	Combination treatment with anti-CTLA4, anti-OX40 and radiation resulted in significantly extended survival.

We observed a correlation between baseline PD-L1 expression of myeloid cells (Fig. 4f) but not CD45-negative cells (Fig. 4g) and response to ICB. This is an important observation seeing as inclusion criteria for some ICB treatment and/or clinical trials require PD-L1 expression (NCT03258788, NCT02536794). NanoString gene expression analysis of the 1E6 tumors also revealed elevated levels of both CD274 (PD-L1) and CTLA-4 (Fig. 5e), which corresponds with the response to anti-CTLA-4 and anti-PD-L1 monotherapy (Fig. 6a). A limitation to this work is that the TME was not assessed after ICB, which may reveal additional changes to the TME that correlate with response to therapy.

## Conclusion

The evasion of immune surveillance is a challenge in breast ICB therapy that warrants further investigation. Mechanistic understanding of how the TME promotes tumor progression will be critical to understanding which cell populations play the most influential role in promoting an immune escape. However, comprehensive immunophenotyping and response to ICB of mouse models are currently lacking. Here, we performed immunophenotyping of the MMTV-PyMT autochthonous model compared to 3 versions of syngeneic models derived from MMT-PyMT tumors and tested their response to anti-CTLA4 and anti-PD-L1 therapy. We have revealed that the TME of tumors from the 1E6 and 1E5 syngeneic models are vastly different from the 1E4 and autochthonous models, which was confirmed using 1E6 and 1E4 versions of the EMT6 model. We have uncovered that innate immunity and antigen processing may play a vital role in determining response to checkpoint blockade yet may be an artificial response in the ICB-sensitive models (1E6, 1E5). Further work to characterize the signals within the TME that promote immune evasion will be vital to advancing checkpoint blockade therapy for the treatment of breast cancer. Shedding light on why the ICB-sensitive models are sensitive to ICB therapy and providing syngeneic models to study ICB resistance is a major advancement for the study of immunotherapy in breast cancer and represents a unique opportunity to further interrogate biomarkers of response to ICB.

## Supplementary information

**Additional file 1: Supplemental Figure 1.** Immunophenotyping of cells used for generation of syngeneic murine models. Tumors from the MMTV-PyMT autochthonous model were used to generate inoculum to inject into wild type mice to generate 1E6, 1E5 and 1E4 syngeneic tumor models. Flow cytometry was performed on three separate batches of inoculum, which were used for each unique experiment and are shown as red (inoculum1), blue (inoculum2), and green (inoculum3). a. Gating strategy for flow cytometry b. Immune cell composition as a frequency of immune cell populations out of CD45+ cells. c. Shown are graphs corresponding to Fig. 1e (CD45), Fig. 1g (CD11b) and Fig. 1h (CD3) color coded by inoculum (experimental run). Graphs show mean ±SEM. *P<0.05, **P<0.01, *** P<0.001, **** P<0.001. **Supplemental Figure 2.** Example of FACs gating strategy. Gating strategy for flow cytometry. **Supplemental Figure 3.** Ratio of anti-tumor to pro-tumor macrophages. Tumors from the autochthonous MMTV-PyMT model were harvested and single-cell suspensions were generated. Cells (1E6, 1E5 or 1E4) were injected into mammary fat pad number 4 of FVB/NJ wild type mice. When the tumors reached 100 mm3, tumors were obtained and processed into single-cell suspension for immunophenotyping by flow cytometry to identify the ratio of classically activated macrophages CD40+ (a), CD80+ (b), and CD86+ (c) of F4/80+ to alternatively activated macrophages indicated by the mannose receptor, CD206 + of F4/80 + cells. Each data point represents an individual mouse. Graphs show mean ±SEM. *P<0.05, **P<0.01, *** P<0.001, **** P<0.001. **Supplemental Figure 4.** EMT6 1E6 and 1E4 models reproduce observations from the MMTV-PyMT syngeneic model. Balb/c mice were injected with 1E6 or 1E4 tumor cells in the mammary fat pad. When tumor reached 100-150 mm3, mice were sacrificed, and tumors were obtained for immunophenotyping analysis by flow cytometry. (a) The 1E6 tumors reached 100-150 mm3 faster than the 1E4 tumors. (b) Batch controls were used to control for variability between running flow experiments on different days, when tumors reached 100-150 mm3. (c-e) Flow cytometry was performed. **Supplemental Figure 5.** QC of mRNA expression analysis. Tumors from the autochthonous MMTV-PyMT model were harvested and single-cell suspensions were generated. Cells (1E6, 1E5 or 1E4) were injected into mammary fat pad number 4 of FVB/NJ wild type mice. When the tumors reached 100 mm3, RNA was extracted from tumors and NanoString analysis was performed. Data was analyzed using the Advanced Analysis 2.0 Module of the nSolverTM software (NanoString Technologies). The geNorm algorithm was used for selecting housekeeping genes and samples were normalized against positive controls. a. Normalization results shows consistency among selected housekeeping genes according to geNorm. b. Mean gene expression plotted versus variance. Housekeeping genes used in normalization are highlighted. c. Principle component analysis scored across the four models. The first two principal components explain 36% and 14% of variance respectively. d. Unsupervised hierarchical clustering was performed on all normalized transcripts above background. Orange cells indicate higher than average expression; blue cells indicate lower than average expression. e. A summary of cell types scores are shown across the four groups. e Quantification of cell types scores for neutrophils, dendritic cells, natural killer cells, and natural killer CD56dim are shown. Each data point represents an individual mouse. Graphs show mean ±SEM. Unpaired two tailed t-test. *P<0.05, **P<0.01, *** P<0.001, **** P<0.001. **Supplemental Figure 6.** Quantification of pathway analysis by NanoString. Signature scores from the NanoString pathways analysis are shown. Graphs show mean ±SEM with 4-5 mice per group. Unpaired two tailed t-test. *P<0.05, **P<0.01, *** P<0.001, **** P<0.001. **Supplemental Figure 7.** Pathway analysis by NanoString. a. The heatmap summarizes the mRNA transcripts categorized by their respective pathway. Shown are the signature scoring differences of the syngeneic models compared to the transgenic model. Orange represents higher than average scores; blue represents lower than average scores. b-d. Volcano plots representing differential expression analysis of genes expressed in the 1E6 (top), 1E5 (middle), 1E4 model (bottom) compared to the transgenic model. Volcano plots show fold change (log2) versus log10 p-values. False discovery rate thresholds are shown (FDR<0.01, FDR<0.05, FDR<0.1, and FDR<0.5). e. Normalized log2 counts of transcripts related to the Antigen presentation pathway from the Nanostring pathways analysis are shown. f. Normalized log2 counts of transcripts related to the immune signaling pathway are shown. Graphs show mean ±SEM with 4-5 mice per group. Unpaired two tailed t-test. *P<0.05, **P<0.01, *** P<0.001, **** P<0.001. **Supplemental Figure 8.** Syngeneic tumor treated with CTLA-4 and PD-L1 show significant differences in tumor responses. Mice of similar tumor burden from each syngeneic model and the autochthonous model were randomized into four treatment groups: IgG2B, PD-L1 inhibitor, CTLA-4 inhibitor, and the combination of PD-L1 and CTLA-4 inhibitors. Tumors were measured every 2-3 days. Tumor volumes were measured and plotted as mean total tumor burden ± SEM and tumor burden fold change were calculated at day 14 for the 1E6 (a), 1E5 (b), 1E4 (c) and autochthonous (d) models. MMTV-PyMT autochthonous mice were enrolled into as study at about 80 days old and when each tumor reached 80-100 mm3. Mice have 10 mammary fat pads and tumor arise in all 10 fat pads. Tumors from mammary fat pad numbers 5 and 10 were excluded from our analysis. The sum of the volumes for tumors from the MMTV-PyMT autochthonous fat pads (1-4 and 5-9) were used and indicated as “total tumor burden”. The syngeneic mice that had tumors that measured 80-100 mm3 were enrolled into an experiment. Shown are a summary of two individual experiments n=3-7/treatment groups. Two-way ANOVA multiple comparisons was performed *P<0.05, **P<0.01, *** P<0.001, **** P<0.001. **Supplemental Figure 9.** Comparison of predicted responders vs. non-responders by NanoString analysis. The NanoString data was reanalyzed grouping mice into predicted responders (1E6 and 1E5) and predicted non-responders (1E4 and autochthonous). a. Principle component analysis. b. Heatmap of immune cell scores. c. Cell types scores are shown across the two groups. Each data point represents an individual mouse. d. Heatmap of pathway analysis showing predicted responders (gray) and non-responders (orange). e. Differential expression of responders vs non-responders with the Inflammation Score Probe Set shown in red. Graphs show mean ±SEM. Unpaired two tailed t-test. *P<0.05, **P<0.01, *** P<0.001, **** P<0.001. **Supplemental Figure 10.** Summary slide of cellular immune components that correlate with response to immunotherapy. a. Key characteristics of immune cell subsets are summarized in the table and grouped by T-cells, myeloid cells, where “+” indicates significant changes, and blue and red represent significant increase and decrease expression, respectively, compared to the MMTV-PyMT model. Response to immune checkpoint blockade is also shown where blue and red represent significant increase and decrease response, respectively, compared to the vehicle control. b. Schematic representing the 4 tumor models and their immune composition. Figure created using biorender.com.

## Data Availability

The datasets used and/or analyzed during the current study are available from the corresponding author on reasonable request.
