# Supplementary material for: Comparing syngeneic and autochthonous models of breast cancer to identify tumor immune components that correlate with response to immunotherapy in breast cancer
Source: Breast Cancer Res. 2021 Aug 5;23:83. doi: 10.1186/s13058-021-01448-1 (PMC8340363; doi:10.1186/s13058-021-01448-1)
Supplement: Supplementary file 1 — Additional file 1: Supplemental Figure 1. Immunophenotyping of cells used for generation of syngeneic murine models. Tumors from the MMTV-PyMT autochthonous model were used to generate inoculum to inject into wild type mice to generate 1E6, 1E5 and 1E4 syngeneic tumor models. Flow cytometry was performed on three separate batches of inoculum, which were used for each unique experiment and are shown as red (inoculum1), blue (inoculum2), and green (inoculum3). a. Gating strategy for flow cytometry b. Immune cell composition as a frequency of immune cell populations out of CD45+ cells. c. Shown are graphs corresponding to Fig. 1e (CD45), Fig. 1g (CD11b) and Fig. 1h (CD3) color coded by inoculum (experimental run). Graphs show mean ±SEM. *P<0.05, **P<0.01, *** P<0.001, **** P<0.001. Supplemental Figure 2. Example of FACs gating strategy. Gating strategy for flow cytometry. Supplemental Figure 3. Ratio of anti-tumor to pro-tumor macrophages. Tumors from the autochthonous MMTV-PyMT model were harvested and single-cell suspensions were generated. Cells (1E6, 1E5 or 1E4) were injected into mammary fat pad number 4 of FVB/NJ wild type mice. When the tumors reached 100 mm3, tumors were obtained and processed into single-cell suspension for immunophenotyping by flow cytometry to identify the ratio of classically activated macrophages CD40+ (a), CD80+ (b), and CD86+ (c) of F4/80+ to alternatively activated macrophages indicated by the mannose receptor, CD206 + of F4/80 + cells. Each data point represents an individual mouse. Graphs show mean ±SEM. *P<0.05, **P<0.01, *** P<0.001, **** P<0.001. Supplemental Figure 4. EMT6 1E6 and 1E4 models reproduce observations from the MMTV-PyMT syngeneic model. Balb/c mice were injected with 1E6 or 1E4 tumor cells in the mammary fat pad. When tumor reached 100-150 mm3, mice were sacrificed, and tumors were obtained for immunophenotyping analysis by flow cytometry. (a) The 1E6 tumors reached 100-150 mm3 faster than the 1E4 tumors. (b) Batch co [file 13058_2021_1448_MOESM1_ESM.pdf]

Lal et al. Figure 1. The number of cancer cells inoculated in pre-clinical models influences tumor growth kinetics as well as the tumor microenvironment.

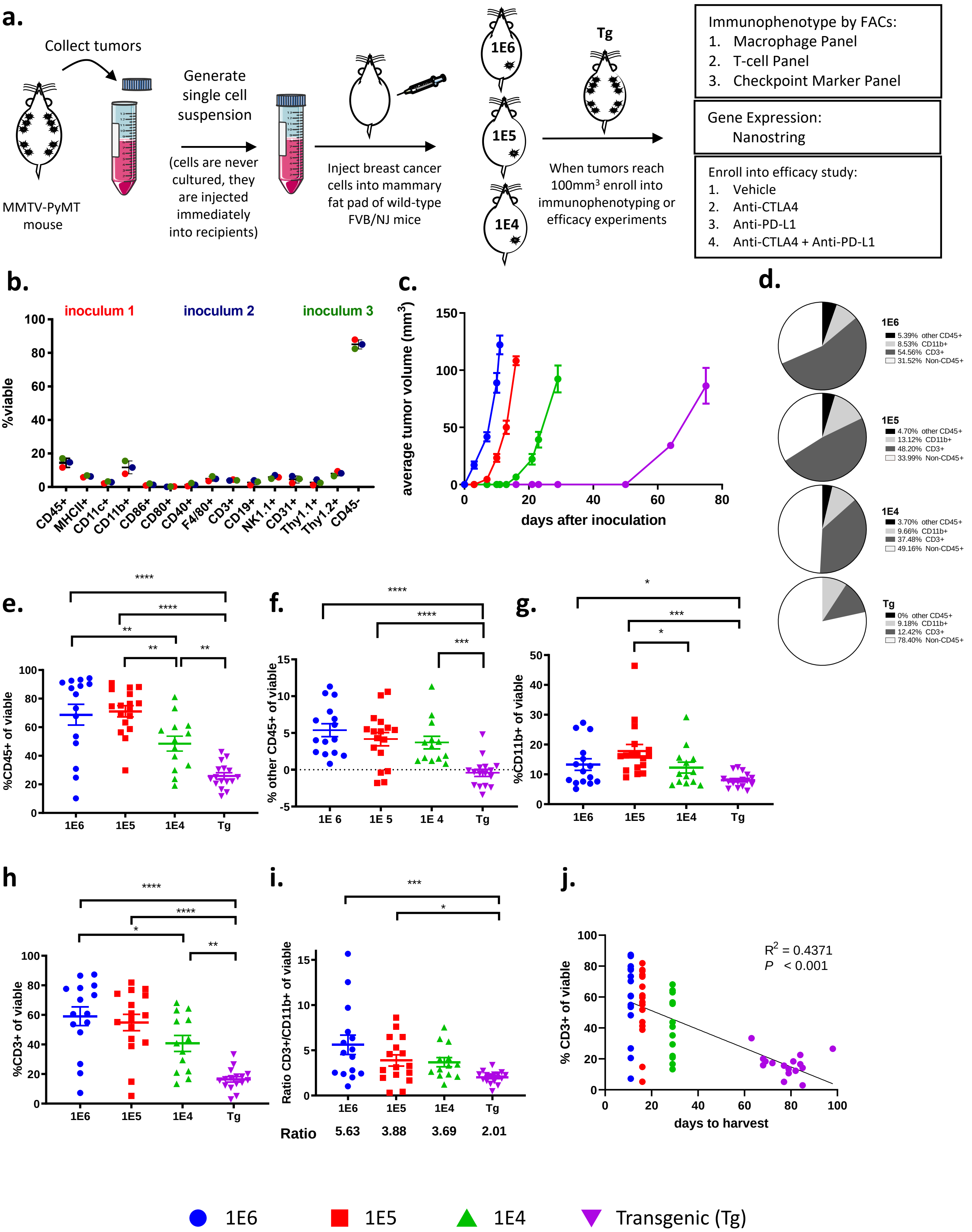

Lal et al. Figure 2. Tumor infiltrating leukocyte populations differ significantly in the different versions of the MMTV-PyMT breast tumor model.

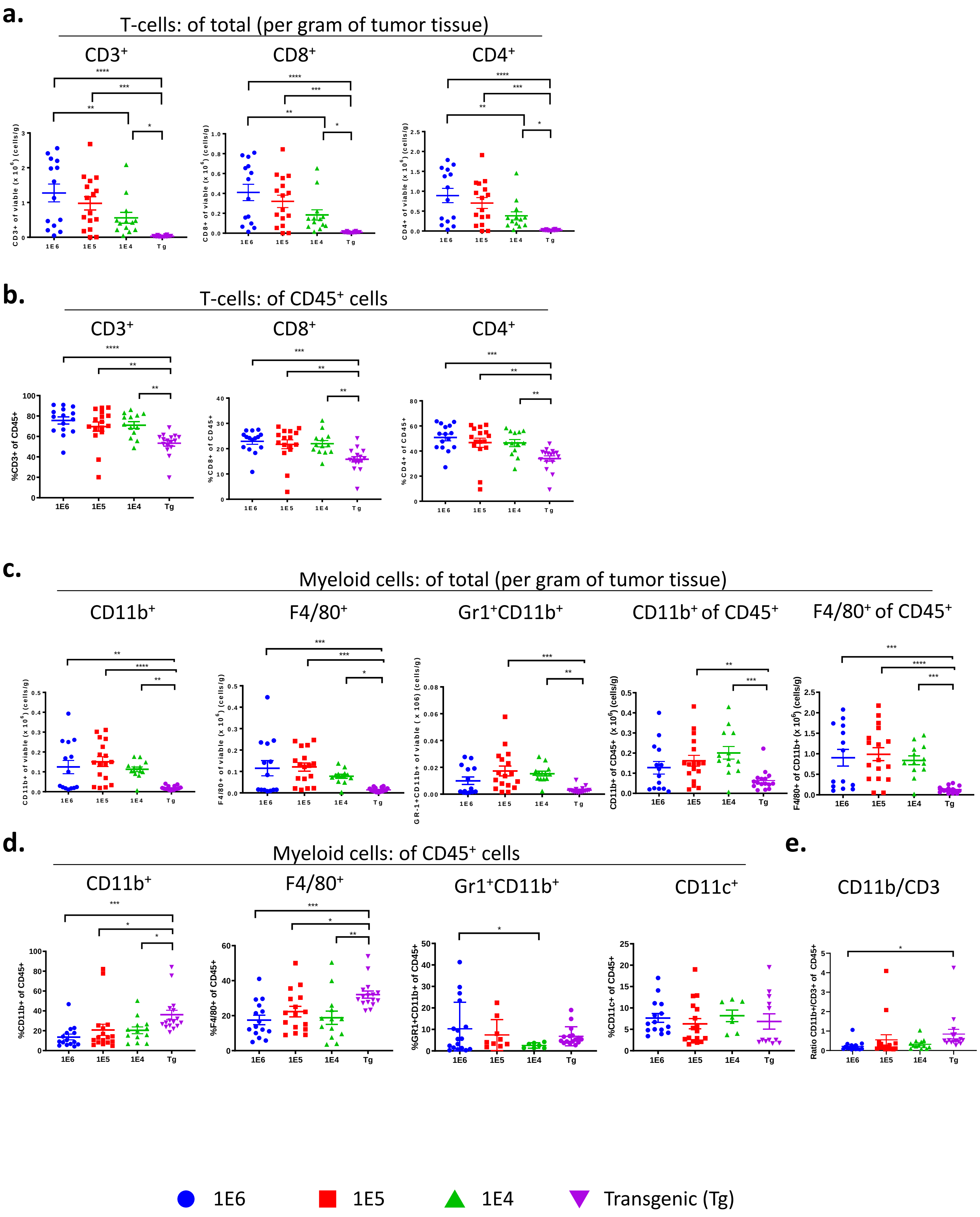

Lal et. al. Figure 3. T-cell immune subsets differ significantly between the different versions of the MMTV-PyMT breast tumor model.

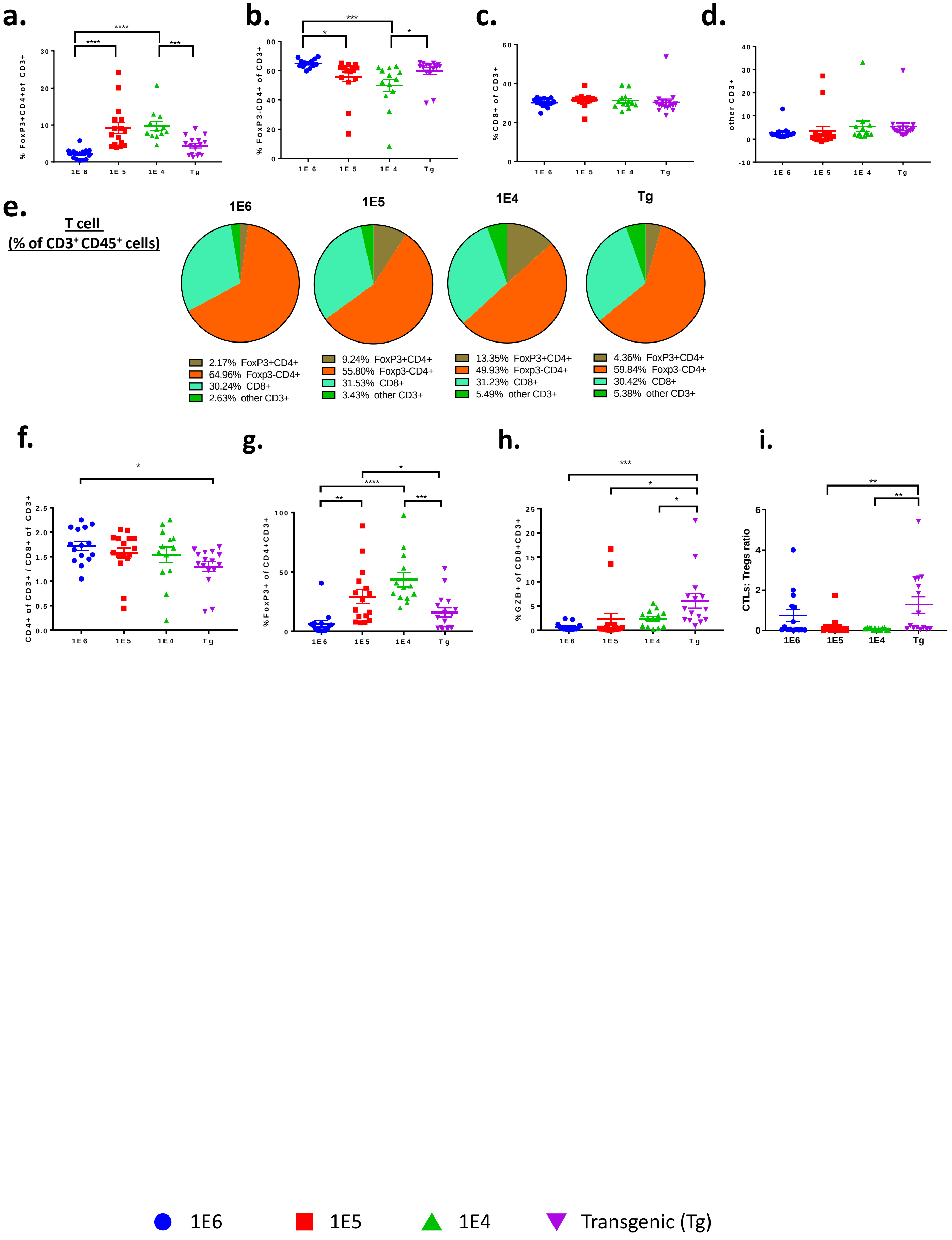

Lal et. al. Figure 4. Myeloid immune cell subsets differ significantly in the different versions of the MMTV-PyMT breast tumor models.

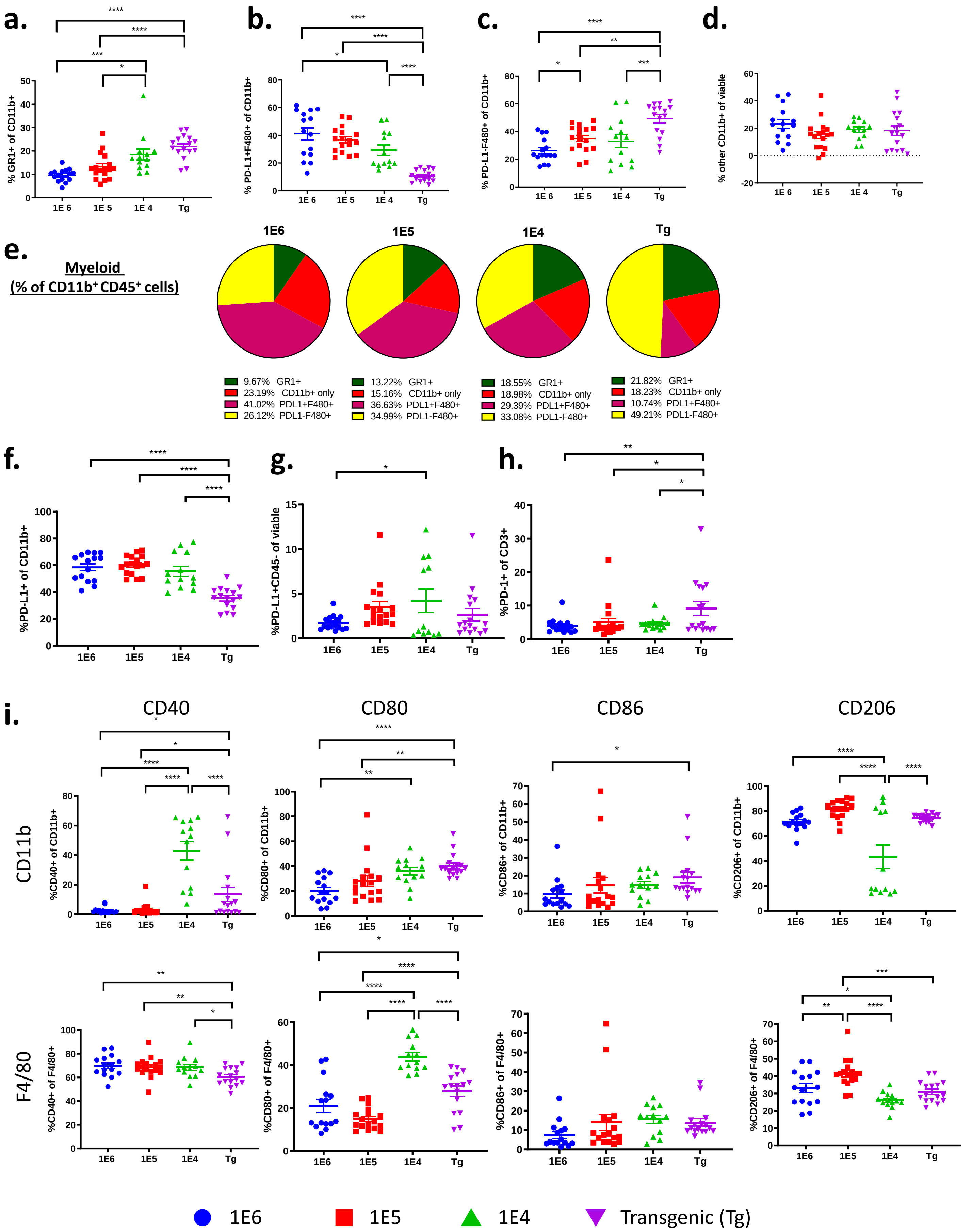

Lal et. al. Figure 5. The different versions of the MMTV-PyMT breast tumor models have distinct immune transcriptional signatures.

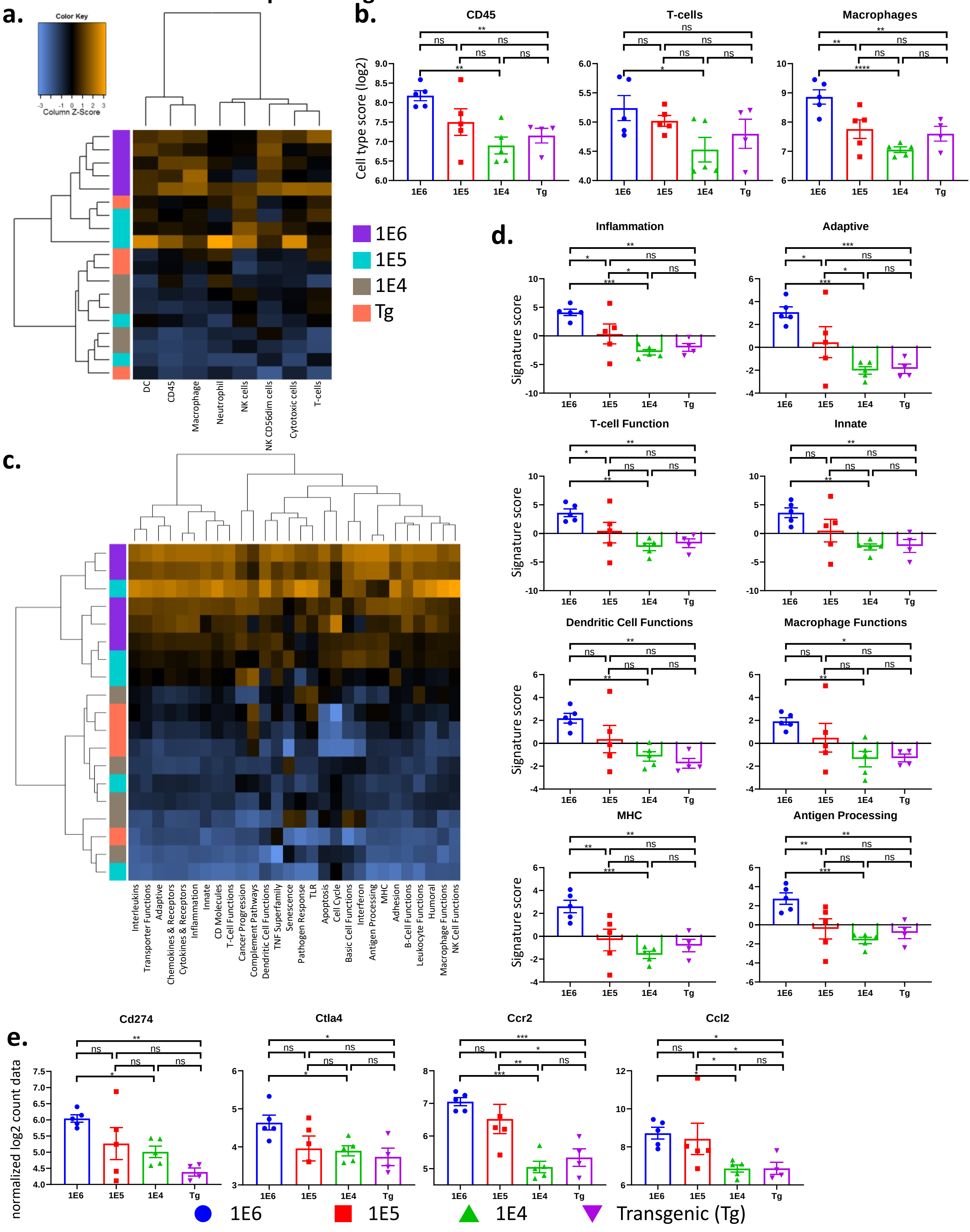

Lal et al. Figure 6. The different versions of the MMTV-PyMT syngeneic model respond differently to immune checkpoint blockade.

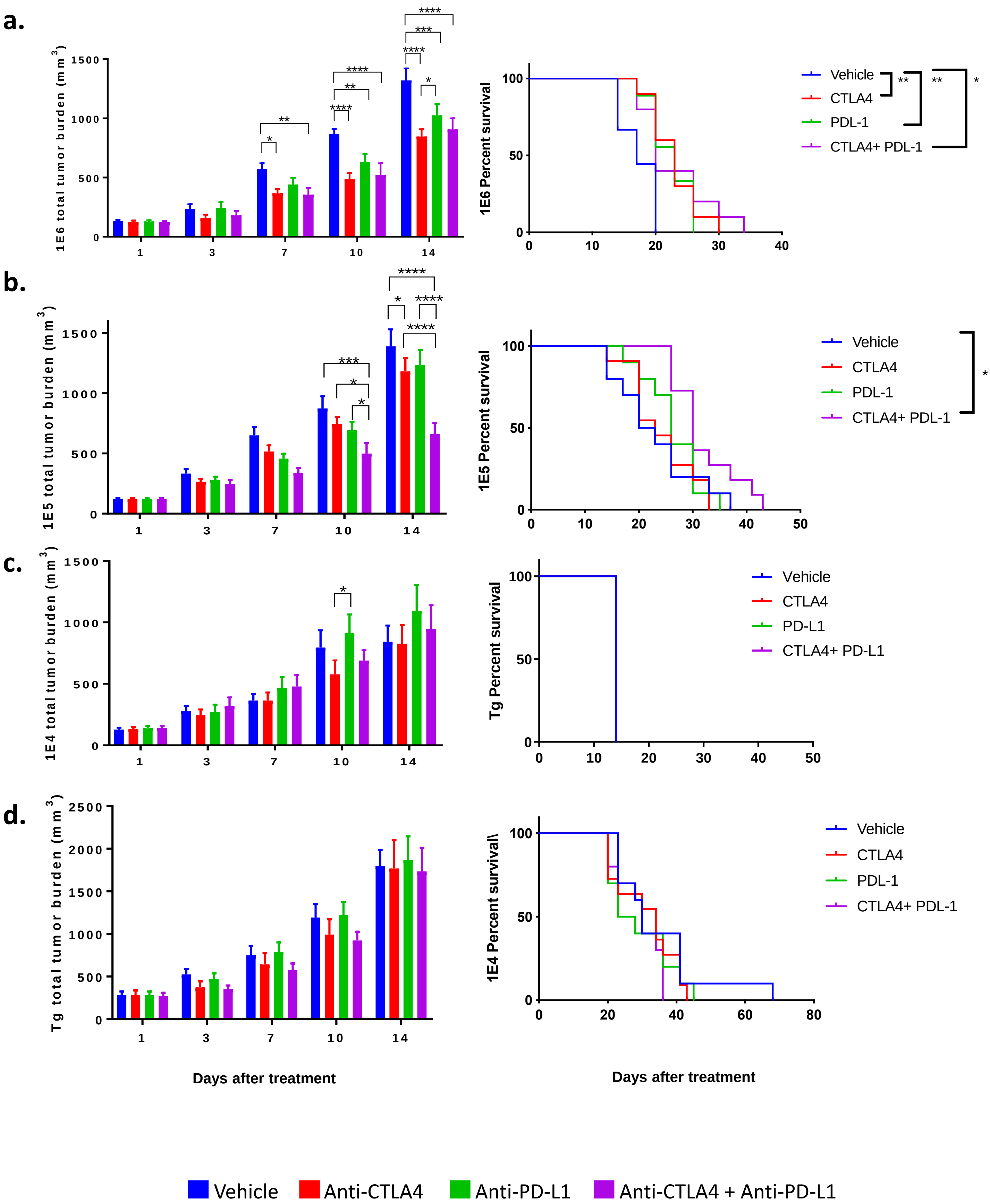

Lal et al. Figure 7. Testing tumor growth kinetics and immunotherapy response in immunodeficient and EMT6 murine models.

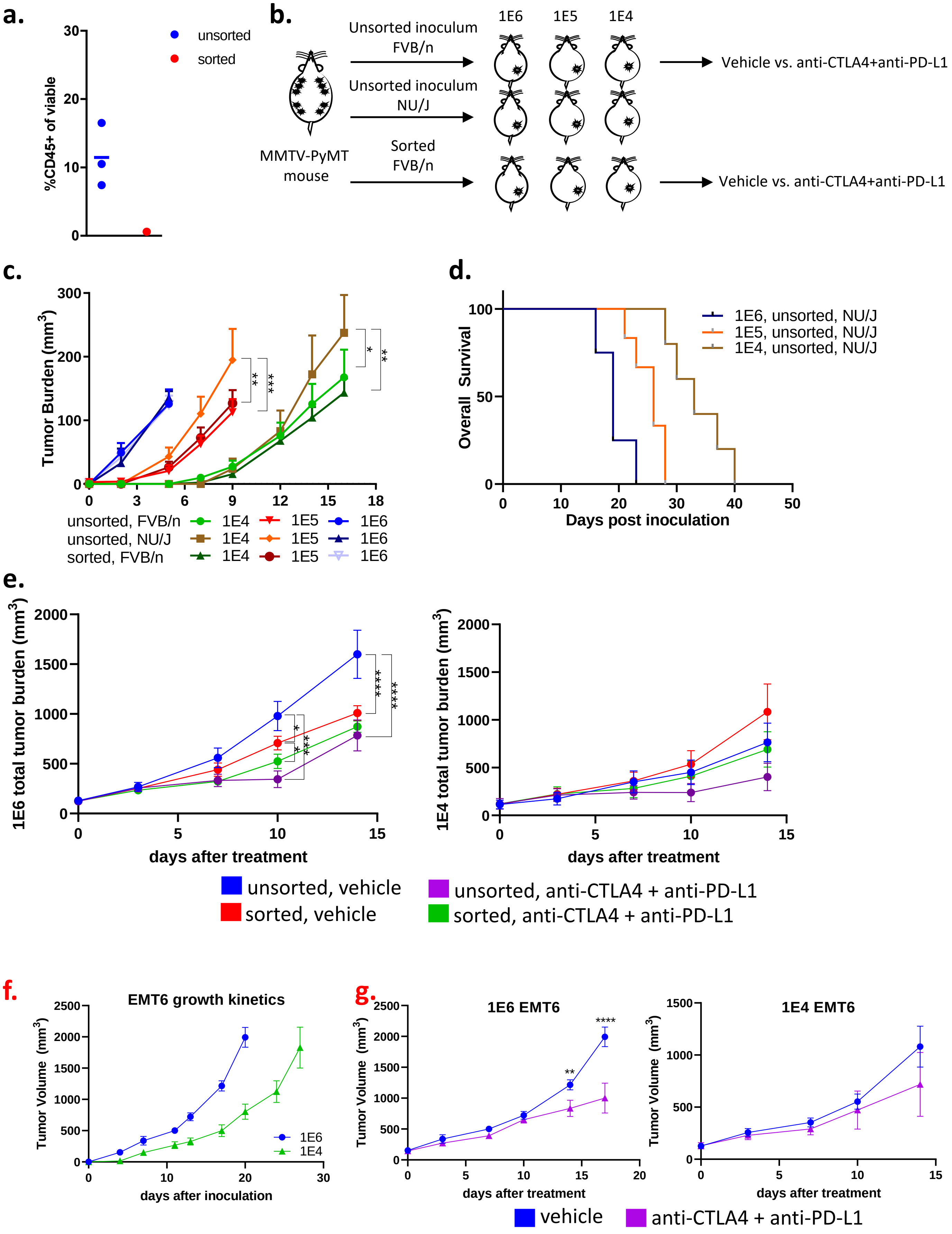

Lal et. al. Supplemental Figure 1. Immunophenotyping of cells used for generation of syngeneic murine models.

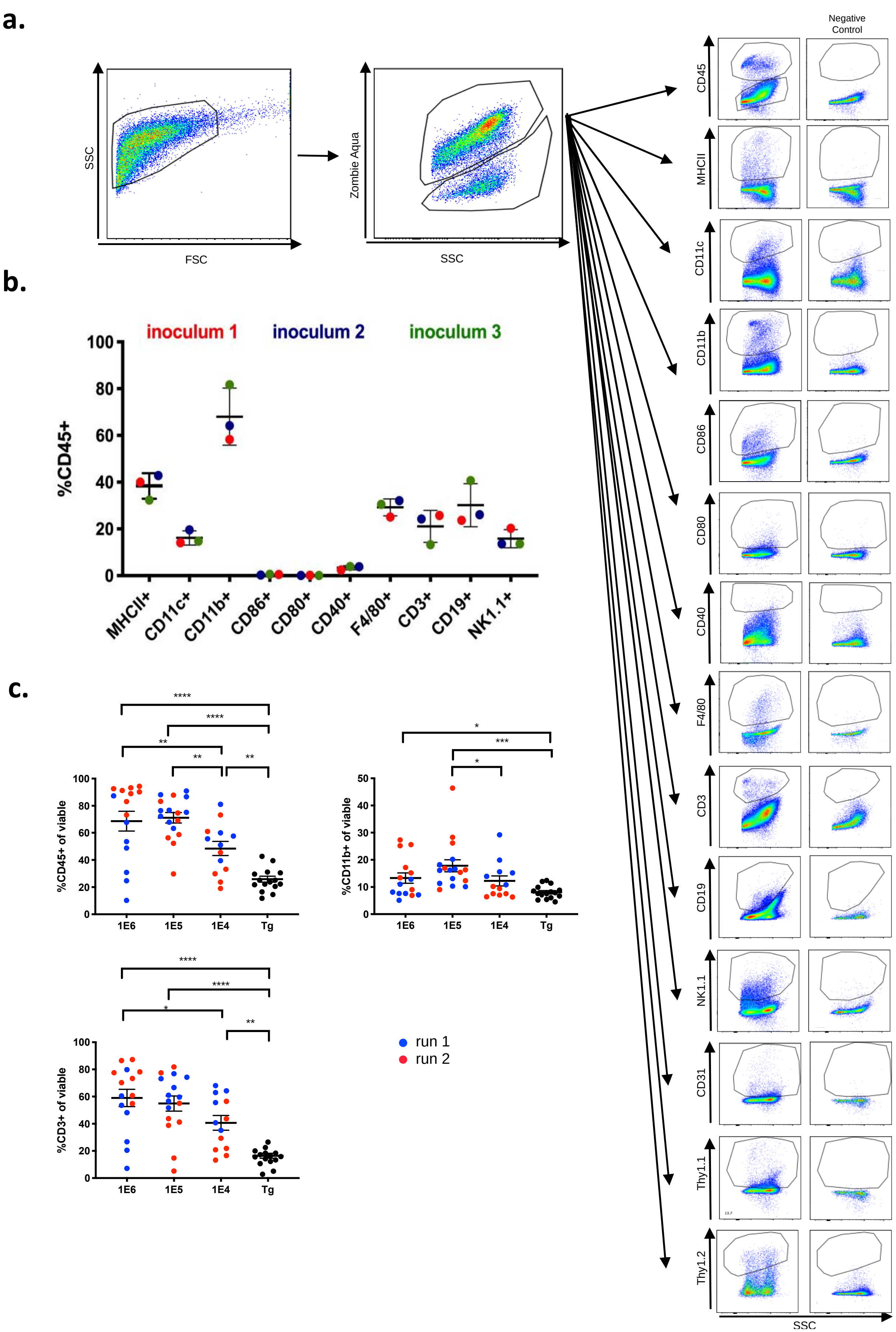

Lal et al. Supplemental Figure 2. Example of FACs gating strategy.

a.

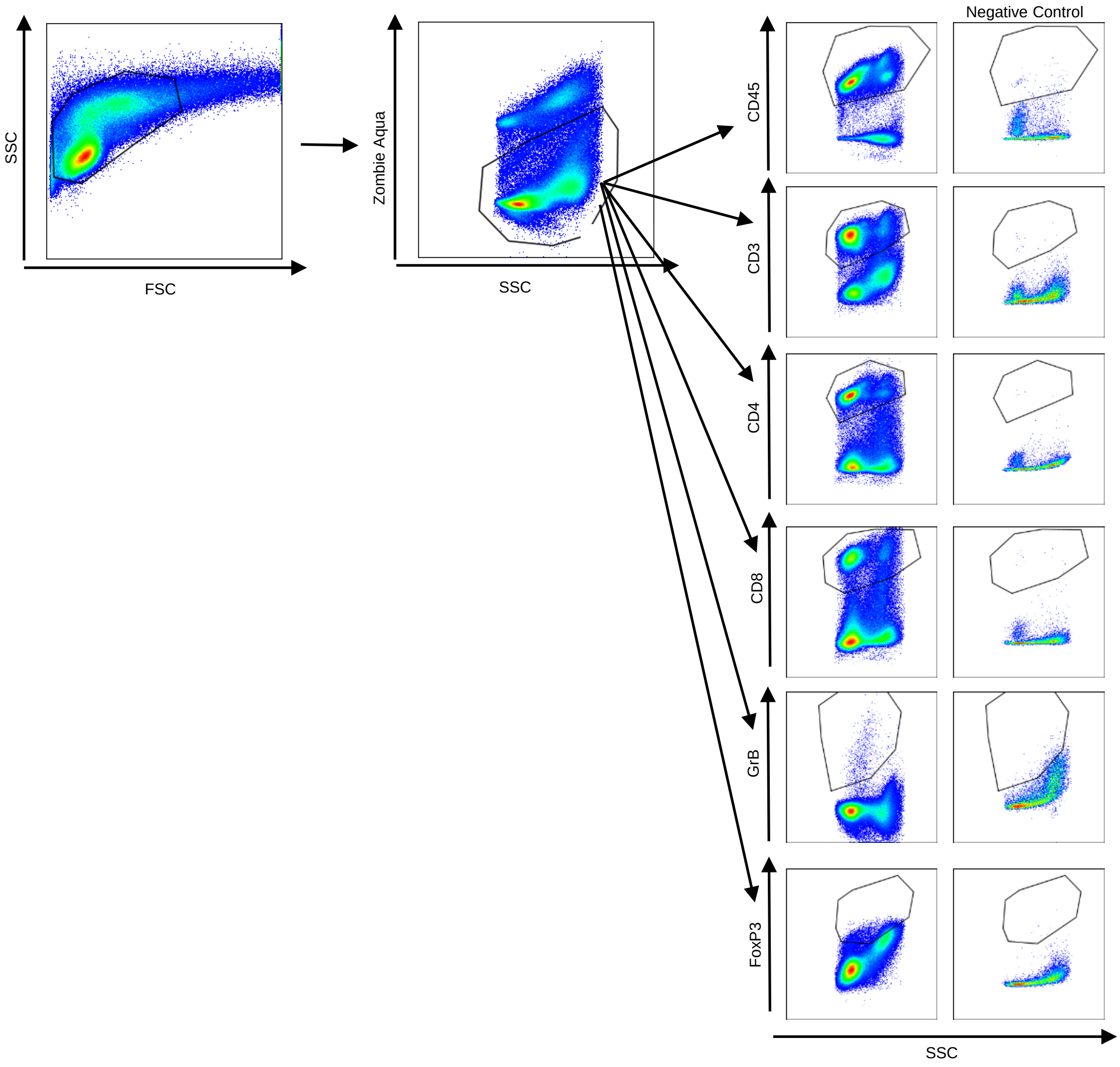

Lal et. al. Supplemental Figure 3. Ratio of anti-tumor to pro-tumor macrophages.

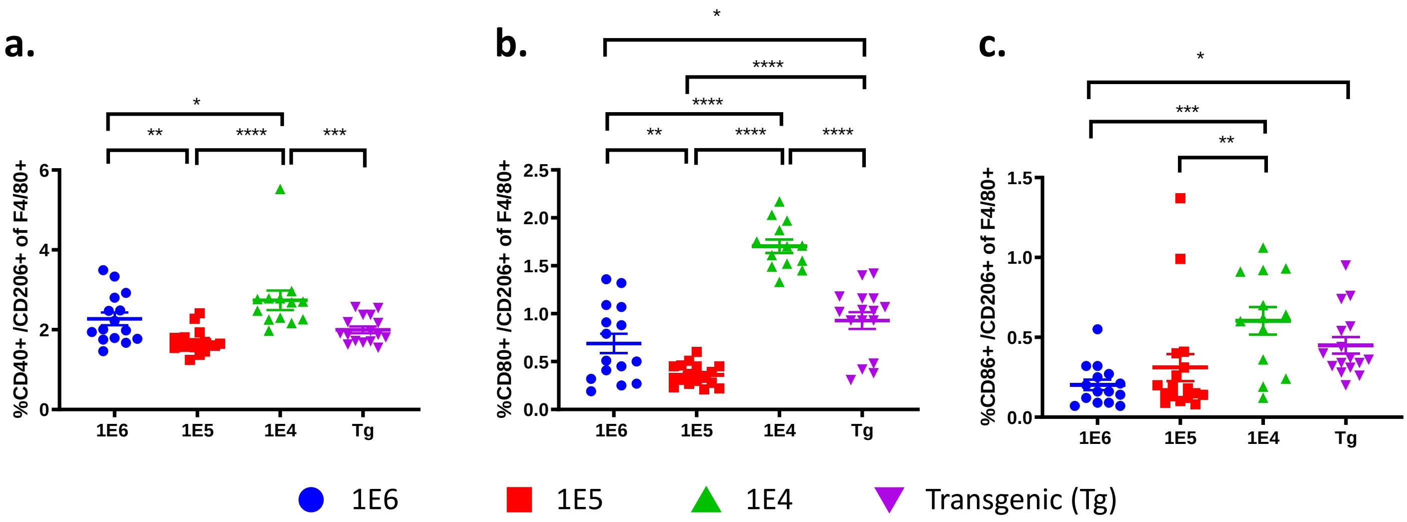

Lal et. al. Supplemental Figure 4. EMT6 1E6 and 1E4 models reproduce observations from the MMTV-PyMT syngeneic model.

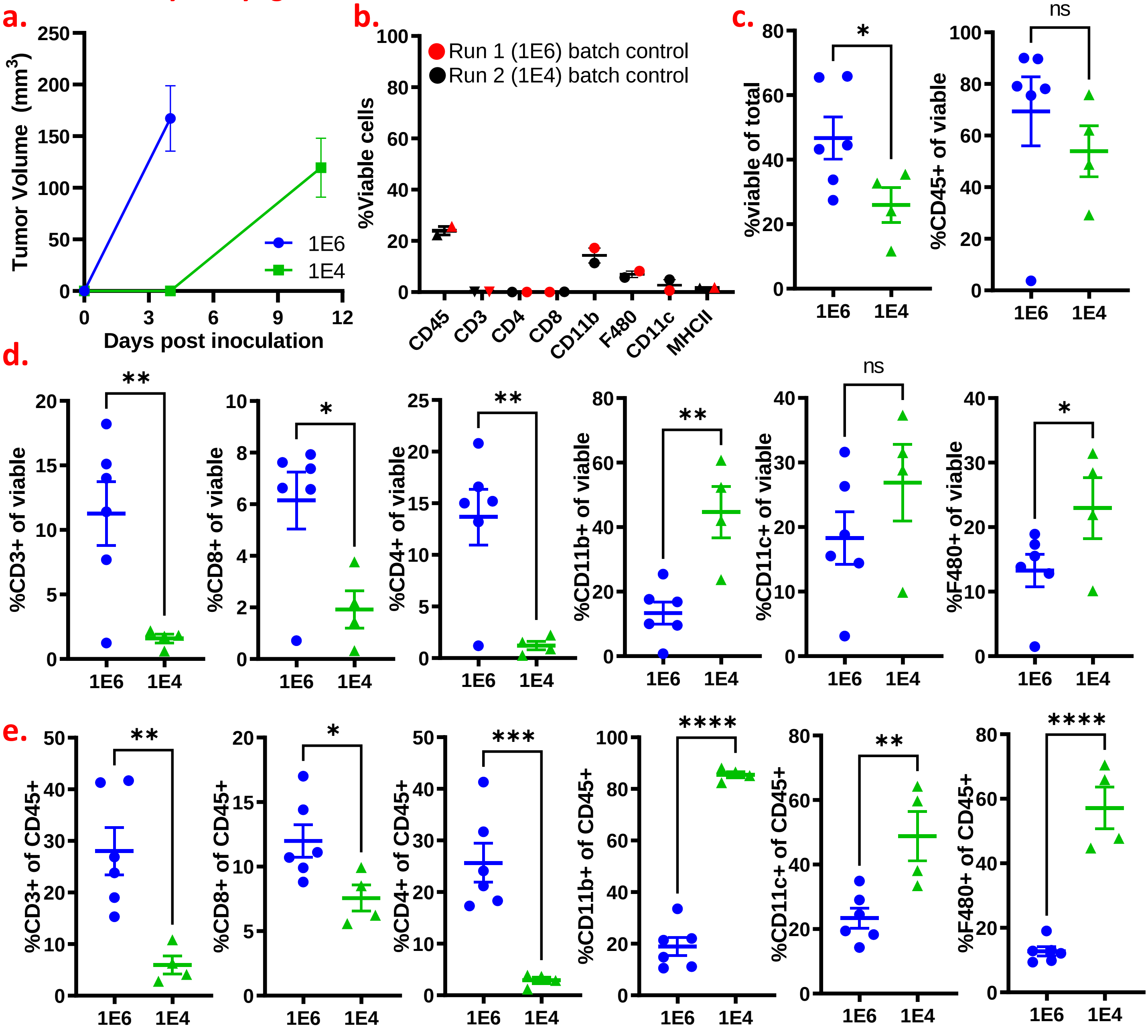

Lal et. al. Supplemental Figure 5. QC of mRNA expression analysis and cell type scores.

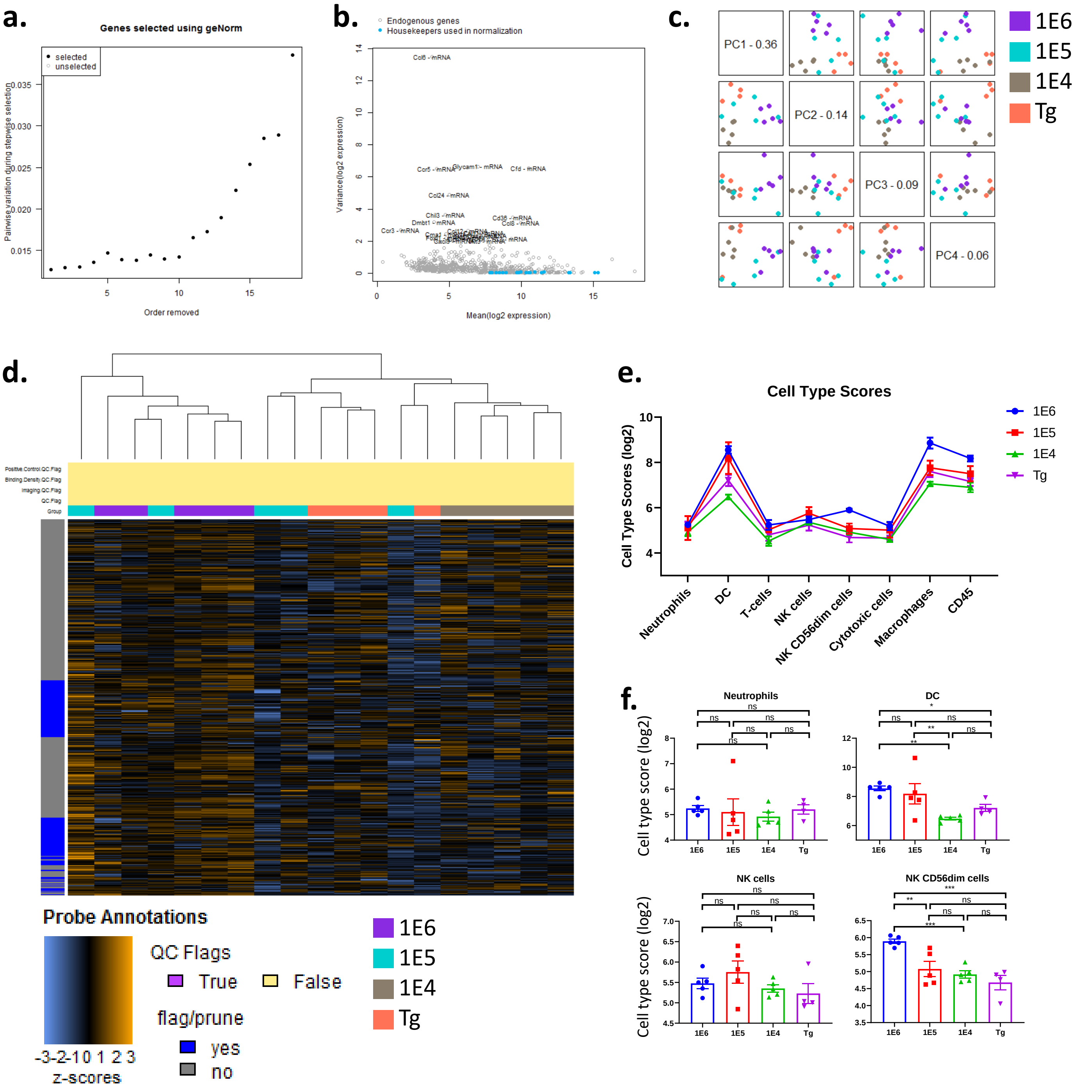

1E6 1E5 1E4 Transgenic (Tg)

Lal et. al. Supplemental Figure 6. Pathway analysis by Nanostring.

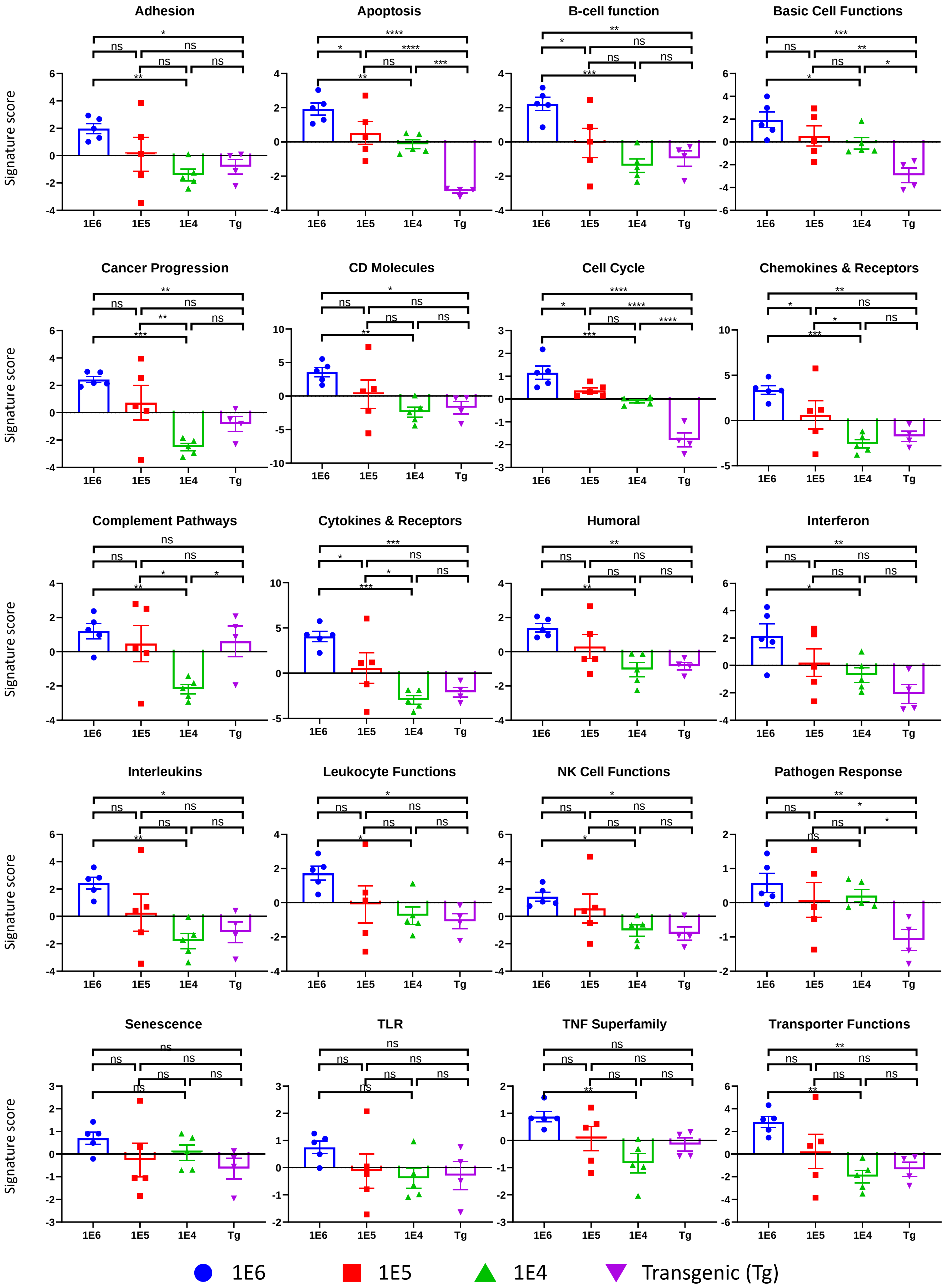

Lal et. al. Supplemental Figure 7. Pathway analysis by NanoString.

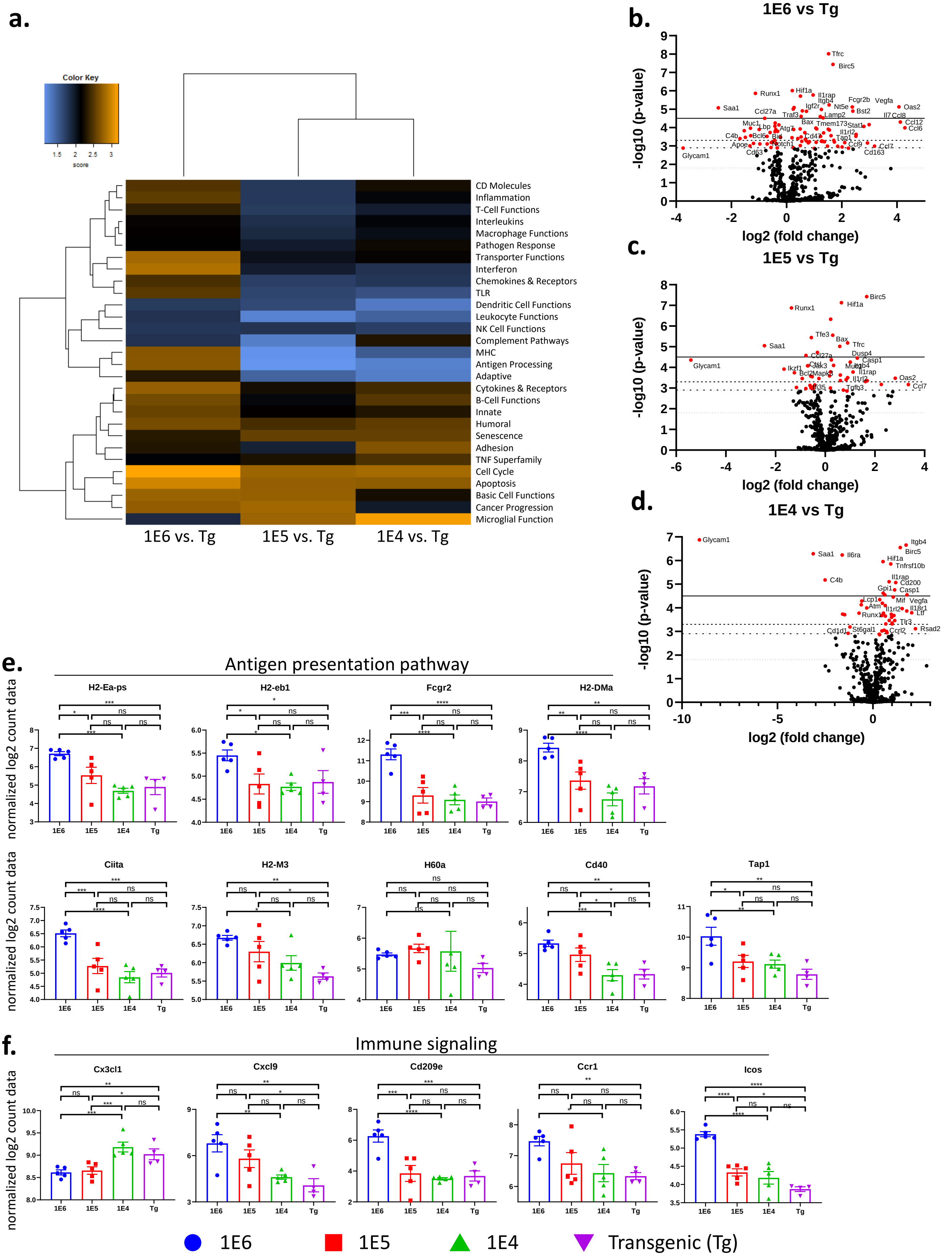

Lal et. al. Supplemental Figure 8. Syngeneic tumors treated with CTLA-4 and PD-L1 show significant differences in tumor responses.

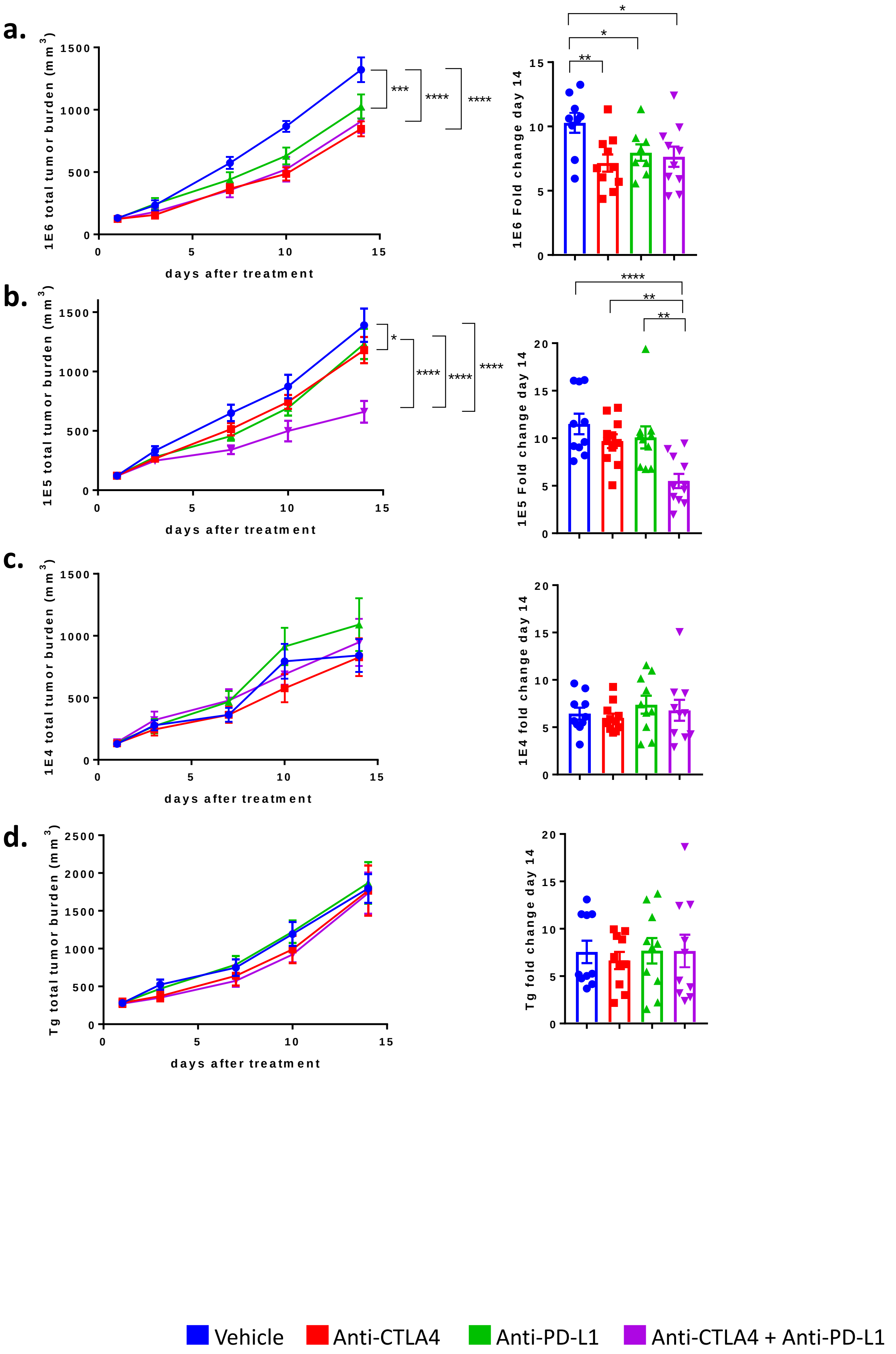

Lal et. al. Supplemental Figure 9. Comparison of predicted responders vs. non-responders by NanoString analysis.

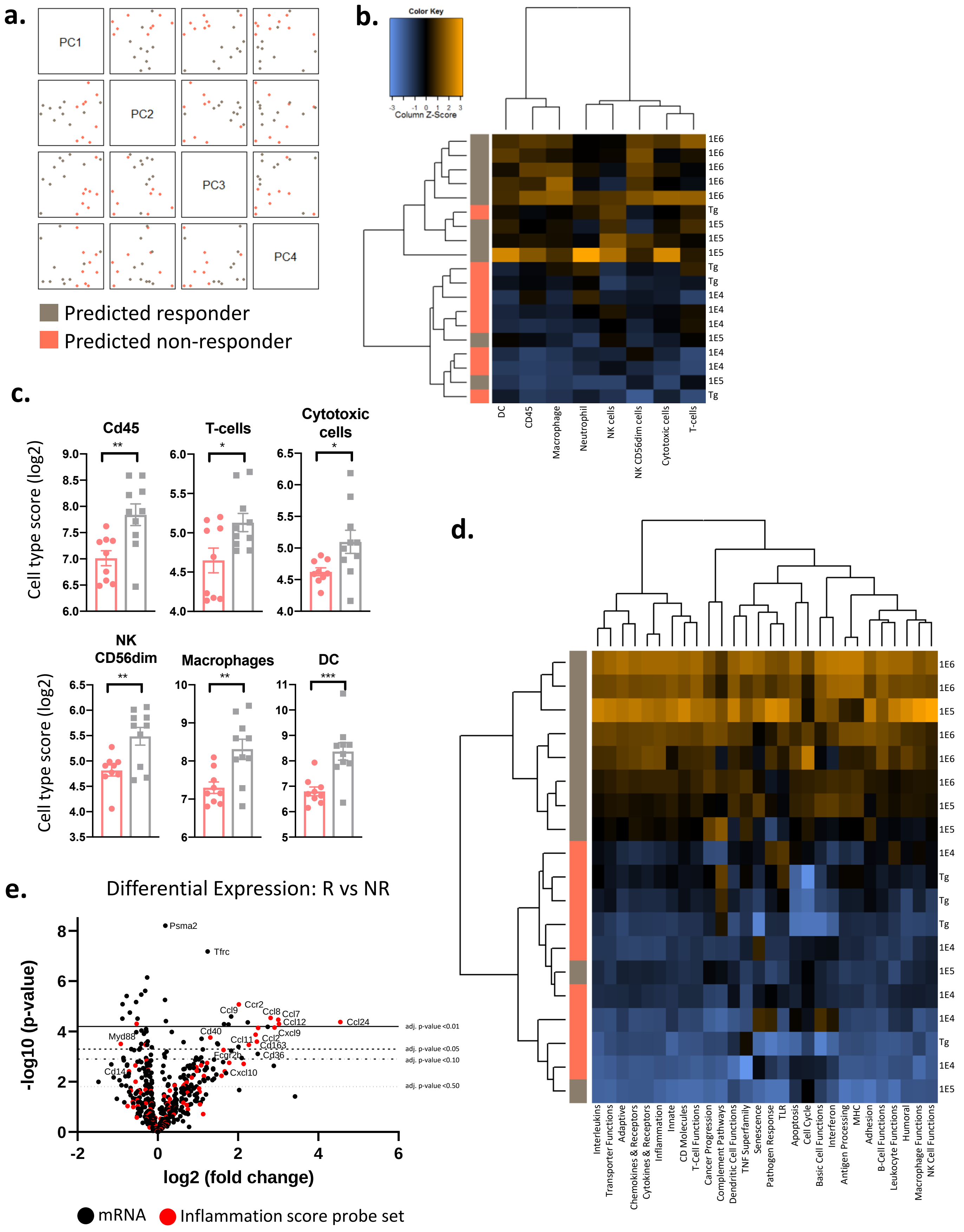

Lal et al. Supplemental Figure 10. Summary Slide.

a.

T cells

	1E6	1E5	1E4
Tumor growth	+++	++	+
CD45+/Viable	+++	++	+
CD3+/viable	+++	++	+
CD8+/viable	+++	++	+
CD4+/viable	+++	++	+
CD11b+/viable	+	+	-
Ratio: CD3+/CD11b of viable	+	+	-
CD3+/CD45+	++	++	++
CD8+/CD45+	+	+	+
CD4+/CD45+	++	++	+
Ratio: CD4+ of CD3+/CD8+ of CD3+	+	-	-
GrB+/CD8+CD45+	-	+	+
FoxP3+/CD4+CD45+	+	+	+
Ratio: CTLs/Tregs	-	+	+
PD-1+/CD3+	+	+	+

+	Significant compared to MMTV-PyMT
-	Not significant compared to MMTV-PyMT
(blue)	Increase compared to MMTV-PyMT
(red)	Decrease compared to MMTV-PyMT

Myeloid Cells

	1E6	1E5	1E4
CD11b+/CD45+	+	+	+
F480+/CD45+	+	+	+
Gr1+CD11b+/CD45+	-	-	-
CD11c+/CD45+	-	-	-
Gr1+/CD11b+	+	+	-
PD-L1+F480+/CD11b+	++	++	+
PD-L1+/CD11b+	+	+	+
PD-L1-F480+/CD11b+	+	+	+
PD-L1+CD45-/Viable	-	-	-
CD40+/CD11b+	+	+	+
CD80+/CD11b+	+	+	-
CD86+/CD11b+	+	-	-
CD206+/CD11b+	-	-	+
CD40+/F480b+	+	+	+
CD80+/F480+	+	+	+
CD86+/F480+	-	-	-
CD206+/F480+	-	+	-
Ratio: M1/M2 macrophages	+	+	+
Antigen processing/MHC	++	-	-
CD11b+/CD3+ of CD45	+	-	-

ICB Efficacy

	1E6	1E5	1E4	Tg
CTLA-4 monotherapy	+	+	-	-
PD-L1 monotherapy	+	-	-	-
PD-L1 + CTLA-4 combination therapy	++	++	-	-

+	Significant compared to Vehicle
-	Not significant compared to Vehicle
(blue)	Increase compared to Vehicle
(red)	Decrease compared to Vehicle

b.

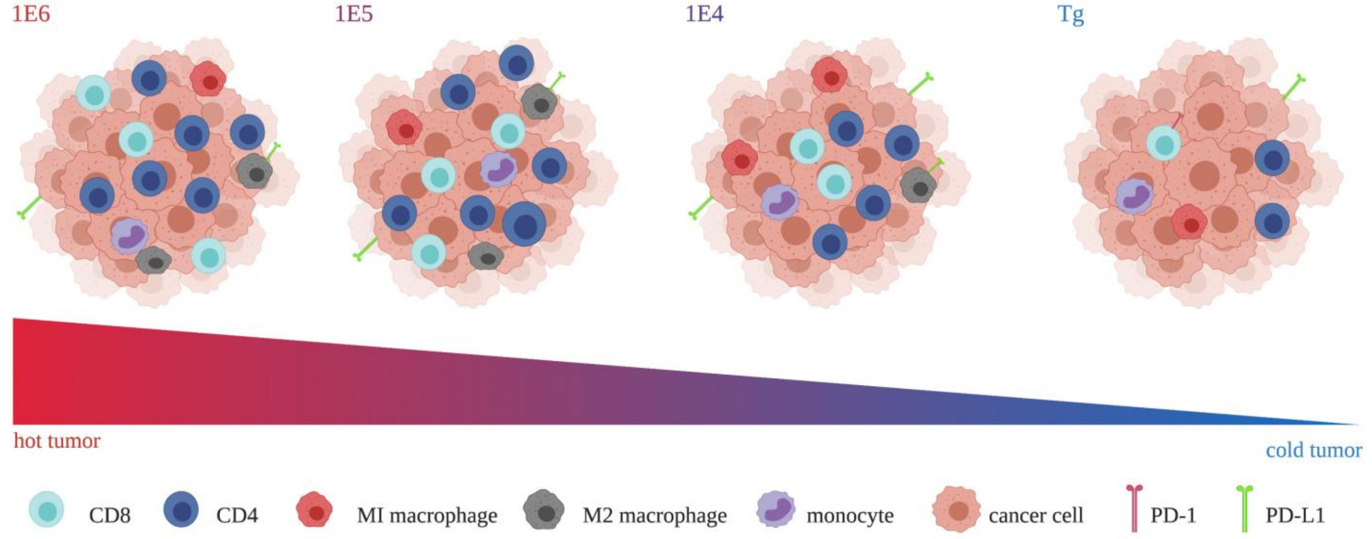

Table 1. List of Top 20 differential expression of immune transcripts in 1E6 versus the autochthonous model.

Transcript	Gene. Sets	probe.ID	Log2 fold change	P-value
Tfrc-mRNA	CD molecules, Transporter Functions	NM_011638.3:1930	1.52	9.64E-09
Birc5-mRNA	Apoptosis, Cell Cycle, Cytokines & Receptors	NM_009689.2:237	1.68	3.66E-08
Psma2-mRNA	Cancer Progression	NM_008944.2:136	0.206	9.74E-07
Runx1-mRNA		NM_001111021.1:3055	-1.13	1.38E-06
Il1rap-mRNA	Cytokines & Receptors, Inflammation, Innate, Interleukins	NM_008364.2:2415	0.961	1.70E-06
Hif1a-mRNA	Apoptosis, Cancer Progression	NM_010431.2:1294	0.503	1.94E-06
Nt5e-mRNA	B-Cell Functions, CD molecules, Inflammation	NM_011851.3:1600	1.54	5.90E-06
Oas2-mRNA	Basic Cell Functions	NM_145227.3:414	4.07	7.48E-06
Fcgr2b-mRNA	Antigen Processing, B-Cell Functions, CD molecules, Inflammation, Interleukins, MHC, Transporter Functions	NM_001077189.1:1225	2.38	7.55E-06
Ltbr-mRNA	Apoptosis, TNF Superfamily	NM_010736.3:1962	0.274	8.11E-06
Saa1-mRNA	Adhesion, Cytokines & Receptors, Innate, Macrophage Functions	NM_009117.3:351	-2.47	8.41E-06
Smn1-mRNA	Cancer Progression	NM_011420.2:390	0.24	9.88E-06
Itgb4-mRNA	Adhesion, CD molecules	NM_001005608.2:3355	1.26	1.04E-05
Bax-mRNA	Apoptosis, Transporter Functions	NM_007527.3:735	0.573	1.24E-05
Bst2-mRNA	CD molecules, Humoral, Innate	NM_198095.2:468	2.4	1.28E-05
Igf2r-mRNA	Apoptosis, CD molecules, Transporter Functions	NM_010515.1:2585	0.723	1.30E-05
Traf3-mRNA	Apoptosis, Cytokines & Receptors, Innate, TLR	NM_001048206.1:6385	0.526	2.45E-05
Ikzf2-mRNA	T-Cell Functions	NM_011770.4:7230	1.22	2.52E-05
Ddx58-mRNA	Innate, Interferon	NM_172689.3:1751	1.32	3.00E-05
Ccl27a-mRNA	Cytokines & Receptors	NM_001048179.1:265	-0.793	3.15E-05

Table 2. List of Top 20 differential expression of immune transcripts in 1E5 versus autochthonous model.

Transcript	Gene.sets	probe.ID	Log2 fold change	P-value
Birc5-mRNA	Apoptosis, Cell Cycle, Cytokines & Receptors	NM_009689.2:237	1.67	3.78E-08
Hif1a-mRNA	Apoptosis, Cancer Progression	NM_010431.2:1294	0.653	7.39E-08
Runx1-mRNA		NM_001111021.1:3055	-1.36	1.32E-07
Psma2-mRNA	Cancer Progression	NM_008944.2:136	0.218	4.69E-07
Ltbr-mRNA	Apoptosis, TNF Superfamily	NM_010736.3:1962	0.301	2.77E-06
Tfe3-mRNA	Humoral	NM_172472.3:2715	-0.56	3.63E-06
Tfrc-mRNA	CD molecules, Transporter Functions	NM_011638.3:1930	0.905	6.57E-06
Saa1-mRNA	Adhesion, Cytokines & Receptors, Innate, Macrophage Functions	NM_009117.3:351	-2.45	8.98E-06
Bax-mRNA	Apoptosis, Transporter Functions	NM_007527.3:735	0.586	9.66E-06
Smad4-mRNA	Cancer Progression	NM_008540.2:2885	-0.314	1.88E-05
Ccl27a-mRNA	Cytokines & Receptors	NM_001048179.1:265	-0.773	2.64E-05
Dusp4-mRNA	Basic Cell Functions, Innate	NM_176933.4:2200	1.29	3.53E-05
Mapk14-mRNA	Innate, Senescence, Transporter Functions	NM_001168513.1:114	0.246	4.33E-05
Glycam1-mRNA	Adhesion	NM_008134.2:124	-5.41	4.39E-05
Casp1-mRNA	Cytokines & Receptors, Innate, Interleukins, Microglial Functions	NM_009807.2:259	1.01	5.58E-05
Tank-mRNA	Basic Cell Functions, Innate	NM_011529.1:491	0.334	8.05E-05
Ctsl-mRNA	Basic Cell Functions	NM_009984.3:45	-0.688	8.31E-05
Jak3-mRNA	B-Cell Functions, Cytokines & Receptors, Innate, Interleukins, T-Cell Functions	NM_010589.5:145	-0.706	8.42E-05
Ikzf1-mRNA	B-Cell Functions, NK Cell Functions, T-Cell Functions	NM_001025597.1:4420	-1.66	1.22E-04
Psmb7-mRNA	Basic Cell Functions	NM_011187.1:184	0.215	1.65E-04

Table 3. List of Top 20 differential expression of immune transcripts in 1E4 versus autochthonous model.

Transcript	Gene sets	probe.ID	Log2 fold change	P-value
Glycam1-mRNA	Adhesion	NM_008134.2:124	-9.09	1.34E-07
Itgb4-mRNA	Adhesion, CD molecules	NM_001005608.2:3355	1.73	2.23E-07
Birc5-mRNA	Apoptosis, Cell Cycle, Cytokines & Receptors	NM_009689.2:237	1.43	2.83E-07
Saa1-mRNA	Adhesion, Cytokines & Receptors, Innate, Macrophage Functions	NM_009117.3:351	-3.13	5.19E-07
Il6ra-mRNA	CD molecules, Chemokines & Receptors, Cytokines & Receptors, Interleukins	NM_010559.2:2825	-1.61	5.86E-07
Hif1a-mRNA	Apoptosis, Cancer Progression	NM_010431.2:1294	0.527	1.12E-06
Tnfrsf10b-mRNA	Apoptosis, CD molecules, TNF Superfamily	NM_020275.3:1625	0.934	1.40E-06
C4b-mRNA	Complement Pathway, Humoral, Inflammation, Innate	NM_009780.2:491	-2.51	6.68E-06
Il1rap-mRNA	Cytokines & Receptors, Inflammation, Innate, Interleukins	NM_008364.2:2415	0.844	7.95E-06
Cd200-mRNA	CD molecules	NM_010818.3:686	1.19	8.63E-06
Casp1-mRNA	Cytokines & Receptors, Innate, Interleukins, Microglial Functions	NM_009807.2:259	1.13	1.76E-05
Gpi1-mRNA	Apoptosis, Cytokines & Receptors, Humoral	NM_008155.4:1540	0.536	2.43E-05
Mif-mRNA	B-Cell Functions, Cytokines & Receptors, Inflammation, Innate, Transporter Functions	NM_010798.2:373	0.623	2.84E-05
Vegfa-mRNA	Apoptosis, Cytokines & Receptors, Macrophage Functions, T-Cell Functions	NM_001025250.3:3015	1.78	2.85E-05
Kit-mRNA	CD molecules, Cytokines & Receptors	NM_001122733.1:4275	1.06	3.52E-05
Tank-mRNA	Basic Cell Functions, Innate	NM_011529.1:491	0.353	4.55E-05
Lcp1-mRNA	T-Cell Functions, Transporter Functions	NM_001247984.1:3344	-0.587	5.19E-05
Bax-mRNA	Apoptosis, Transporter Functions	NM_007527.3:735	0.491	6.44E-05
Atm-mRNA	Apoptosis, B-Cell Functions, Cell Cycle, Senescence	NM_007499.2:5543	-0.616	7.50E-05
App-mRNA	Apoptosis, Cell Cycle, Innate, Transporter Functions	NM_007471.2:511	0.641	8.04E-05

Table 4. Published studies on immunotherapy response in syngeneic murine models.

Reference	Model	No. tumor cells inoculated	Tumor size (mm^3)	Time to start treating (d)	Outcome
Kim et al. *PNAS* 2014	4T1	5.00E+06	400	11	Tumor eradication with PD-1/CTLA4 at day 15.
	CT26	5.00E+06	400	11	Tumor eradication with PD-1/CTLA4 at day 25.
Lian et al. *Sci Rep* 2019	4T1	1.00E+05	not reported	24	PD-L1/CD74 treatment, dual blockade reduced lung metastasis.
Clift et al. *Cancer Res* 2019	4T1/EMT6	1.00E+05	100-150	not reported	PD-L1 blockade significantly inhibited tumor growth when combined with PVHA.
Sun et al. *Mol Cancer Ther* 2020	4T1	1.00E+06	100-150	3	Anti-CTLA-4 and anti-PD-1 in combination promoted the infiltration of T cells.
Xie et al. *J Immunother Cancer* 2018	4T1	5.00E+06	< 200	5,8	AngII blockage enhance tumor sensitivity to checkpoint immunotherapy (ctla4/pd1).
Xu et al. *Clin Cancer Res* 2017	EMT6	5.00E+06	not reported	not reported	NHS-muIL12 and avelumab combination therapy enhanced antitumor efficacy.
Knudson et al. *Oncoimmunology* 2018	EMT6	2.50E+05	50-100	not reported	Bifunctional checkpoint inhibitor of TGFβRII linked to the human anti-PD-L1 heavy chain reduced tumor burden.
	4T1	5.00E+04	50-100	not reported	
Zippelius et al. *Cancer Immunol Res* 2015	EMT6	2.50E+05	not reported	16	Induced PD-L1 Expression Mediates Acquired Resistance to agonistic anti-CD40 treatment.
	MC38	1.00E+06	not reported	16	
Lewis et al. *Oncoimmunology* 2017	EMT6	1.50E+06	not reported	7	IL-21 inhibition with CTLA4 blockade promoted tumor regression.
Li et al. *Cancer Cell* 2018	4T1	5.00E+04	<200	6	A monoclonal antibody targeting glycosylated PD-L1 (gPD-L1) blocks PD-L1/PD-1 interaction and promotes PD-L1 internalization and degradation.
	EMT6	5.00E+04	<200	6	
Liu et al. *Cancer Discovery* 2016	4T1.2	2.00E+04	40-80	16	Neo-adjuvant PD-1/CD-137 therapy had better efficacy than adjuvant .
	4T1.2	5.00E+04	40-80	10	Increase in tumor-specific CD8+ T cells after neoadjuvant anti-PD-1+anti-CD137 therapy.
	E0771	5.00E+04	40-80	16-18	Neo-adjuvant PD-1+CD-137 therapy had better efficacy than adjuvant.
Liu et al. *Oncoimmunology* 2019	E0771	2.00E+05	50	10	Guadecitabine in combination with AIT resulted in prolonged survival in both 4 T1 and E0771 breast cancer models.
	4T1	5.00E+04	50	10	
Kasikara et al. *Cancer Res* 2019	E0771	1.00E+05	not reported	10	Combination of TAM inhibitor (BMS-777607) and anti-PD-1improved tumor efficacy.
Messenheimer et al. *Clin Cancer Res* 2017	MMTV-PyMT	1.00E+06	<50	7	Sequential combination of anti-OX40 and anti-PD-1 increased efficacy
Nolan et al. *Sci Transl Med* 2018	MMMMTV-cre/Brca1$^{fl/fl}$/p53$^{+/-}$TV-cre/Brca	4.00E+04	100	21	Cisplatin treatment combined with dual anti–PD-1and anti–CTLA4 therapy substantially augmented antitumor immunity.
Young et al. *Plos One* 2016	MMTV-PYMT	1.00E+06	not reported	14	Combination treatment with anti-CTLA4, anti-OX40 and radiation resulted in significantly extended survival
